# Functions of the RIP kinase family members in the skin

**DOI:** 10.1007/s00018-023-04917-2

**Published:** 2023-09-09

**Authors:** Corinne Urwyler-Rösselet, Giel Tanghe, Michael Devos, Paco Hulpiau, Yvan Saeys, Wim Declercq

**Affiliations:** 1 Department of Biomedical Molecular Biology, Ghent University, Ghent, Belgium; 2 VIB Center for Inflammation Research, Ghent, Belgium; 3 Present Address: Department of Biology, Institute of Molecular Health Sciences, ETH Zurich, 8093 Zurich, Switzerland; 4 Present Address: Howest University of Applied Sciences, Brugge, Belgium; 5 Department of Applied Mathematics and Computer Science, Ghent University, Ghent, Belgium

**Keywords:** Receptor interacting kinases, Skin, Keratinocyte, Differentiation, Signalling

## Abstract

The receptor interacting protein kinases (RIPK) are a family of serine/threonine kinases that are involved in the integration of various stress signals. In response to several extracellular and/or intracellular stimuli, RIP kinases engage signaling cascades leading to the activation of NF-κB and mitogen-activated protein kinases, cell death, inflammation, differentiation and Wnt signaling and can have kinase-dependent and kinase-independent functions. Although it was previously suggested that seven RIPKs are part of the RIPK family, phylogenetic analysis indicates that there are only five genuine RIPKs. RIPK1 and RIPK3 are mainly involved in controlling and executing necroptosis in keratinocytes, while RIPK4 controls proliferation and differentiation of keratinocytes and thereby can act as a tumor suppressor in skin. Therefore, in this review we summarize and discuss the functions of RIPKs in skin homeostasis as well as the signaling pathways involved.

## The receptor interacting protein kinase family

The human kinome consists of 518 protein kinases that are classified into different groups and families according to similarities in their kinase domain sequences [1]. The family of receptor interacting protein (RIP) kinases (RIPK) are best known for their functions as cellular integrators of inflammatory and cell death signaling pathways. In this review we focus on general functions and basic regulatory mechanisms of RIPKs as well as on their role in skin homeostasis and skin-associated diseases since most of the RIPK family members have a defined role in controlling keratinocyte cell death, differentiation and inflammatory signaling.

**Fig. 1:**
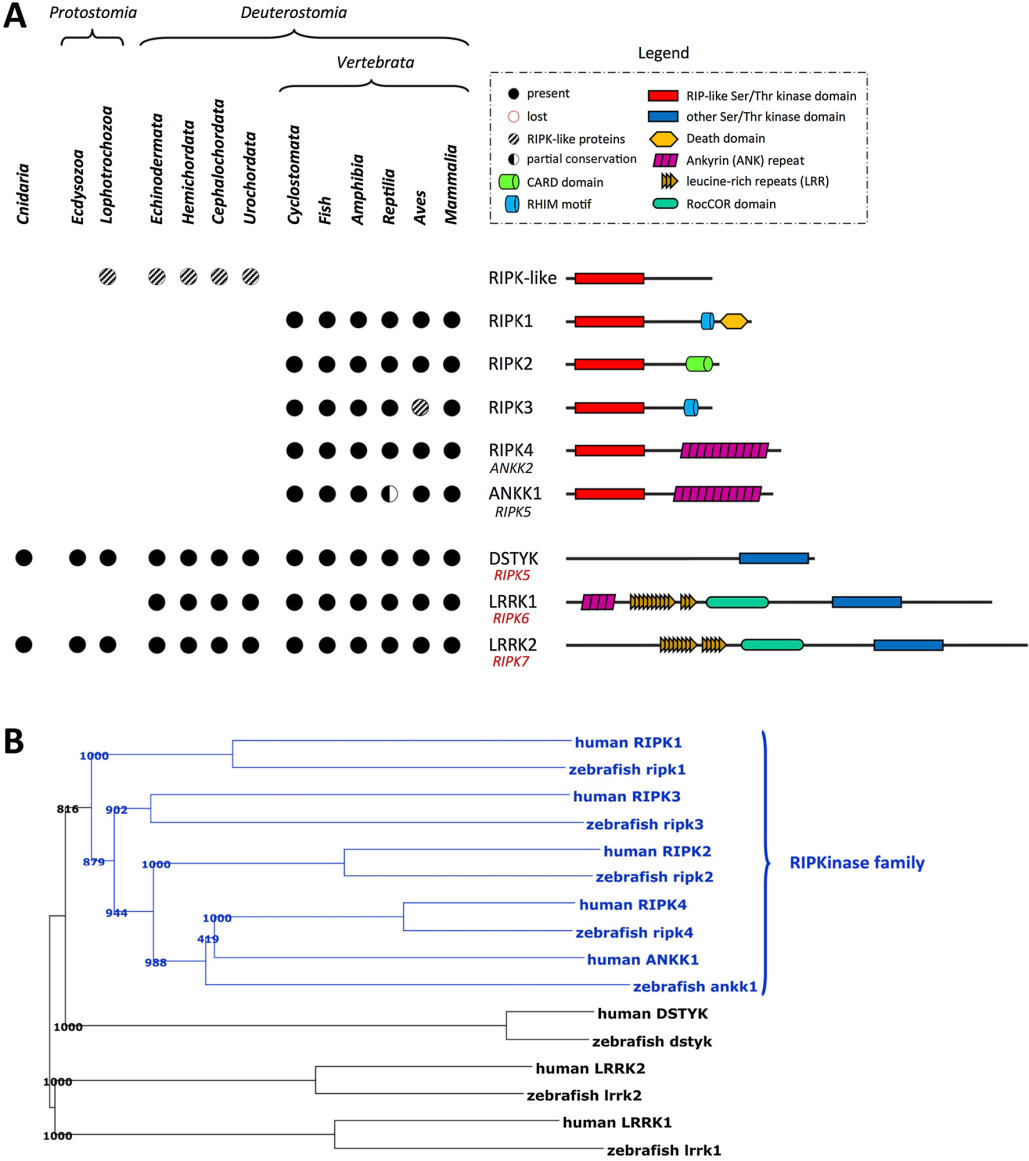
Presence of RIPK family member homologs in the animal kingdom. **A** Evolutionary analysis: publicly available genome assemblies of the indicated lineages were analyzed for the presence or absence of RIPK1, RIPK2, RIPK3, RIPK4, ANKK1 (RIPK5), DSTYK, LRRK1 (RIPK6) and LRRK2 (RIPK7) homologs. In addition, the BLASTP algorithm was used against the predicted proteomes of the indicated lineages. Evaluation of the completeness of the genomic context in ENSEMBL and UCSC genome browsers was combined with validation of the presence/ absence of the RIPK homologs by a BLAST search of conserved sequences against the publicly available genome assemblies. A gene was considered absent, when all of the above-mentioned analysis came out negative. The RIPK-like proteins that are found in *Lophotrochozoa*, *Echinodermata*, *Hemichordata*, *Cephalochordata* and *Urochordata* are quite different from *Vertebrate* RIPK, hence it is possible they are not true RIPK homologs. *RIPK* receptor-interacting kinase,* CARD* caspase recruitment domain,* RHIM* RIP homotypic interaction motif*.* **B** Phylogenetic tree of the kinase domain of RIPK. Zebrafish was selected for a detailed comparative genomics and evolutionary analysis due to its high level of orthology with the human genome at the gene sequence level and its widespread use in the study of vertebrate gene function. The conserved and common kinase domain, which is present in each gene, was utilized to construct a multiple sequence analysis and phylogenetic tree, providing valuable insights into their evolutionary relationship

Based on protein homology analysis, the receptor interacting kinase family has been originally defined as a family containing seven family members [2, 3]. However, defining a protein family through phylogenetic analysis has additional value because it offers a reliable way to investigate the relationship between sequence similarity and function, as well as to trace the evolutionary history of a protein family. Phylogenetic analysis of the kinase domains of RIPKs illustrates the separate evolution of the different RIP kinases, allowing the reliable determination of how different RIPK functions are distributed among distinct clades of the evolutionary tree (Fig. 1). Based on our phylogenetic analysis we suggest there are in fact only 5 genuine RIPKs (1–5) (Fig. 1). DSTYK (confusingly also described as RIPK5), LRRK1 and 2 (respectively described as RIPK6 and -7) are probably not members of the RIPK family. DSTYK was suggested to be a divergent protein kinase which is distant from protein serine/threonine kinases and protein tyrosine kinases [4]. Therefore, in the text, we do not refer to these kinases as RIPKs. Not much is known about DSTYK function, but loss of the kinase domain impaired learning capabilities in mice [5] and it was suggested that DSTYK is involved in human urinary tract development [6]. Intragenic deletions in the DSTYK gene are associated with an autosomal-recessive neurodegenerative subtype of lower limb paralysis with additional diffuse skin and hair dyspigmentation, possibly due to increased melanocyte cell death [7]. Mutations in LRRK1 and 2 both correlate with susceptibility to Parkinson’s disease [8–12], can regulate autophagy [13] and are involved in synaptic vesicle endocytosis [14]. LRRKs belong to the ROCO family of proteins (proteins containing a ROC GTPase and COR domain), which code for large proteins with several domains occurring in prokaryotes and metazoans including plants, Cnidaria, Protostoma or nonvertebrate Deuterostomia (Fig. 1A) [15–17]. In addition, DYSTK, LRRK1 and LRRK2 have a very different domain composition and organization compared to RIPK1-5. Therefore, we conclude that neither DSTYK, which has a dusty (Ser/Thr) kinase domain and not a RIPK kinase domain, nor LRRK1 (RIPK6) and LRRK2 (RIPK7) belong to the RIPK family.

Based on the current genomic data available it seems that one or two ancestral RIP kinase genes arose in the chordate ancestor, which is evidenced by the presence of Ripk1a and Ripk1b in the lancelet Branchiostoma, subphylum Cephalochordata [18]. Phylogenetic analysis suggests Ripk1b is orthologous to the vertebrate RIPK1 [18]. The RIPKs probably arose from an ancestral kinase-domain-only kinase, since RIPK1- and RIPK3-like proteins in the urochordata do not have interaction domains at their C-terminus (Fig. 1A). Hence, the ankyrin repeats present in RIPK4 and RIPK5 were probably acquired after the rise of the first vertebrates by domain shuffling followed by a gene duplication event that resulted in the generation of the two paralogs RIPK4 (ANKK2) and RIPK5 (ANKK1) (Fig. 1b), present on different chromosomes. RIPK3 is lost in birds and RIPK5/ANKK1 is absent in some but not all reptiles, although its paralog RIPK4 is present in both. The Ensembl orthologues data for RIPK3 indicate that for the 27 species of birds and reptiles (*Sauropsida*) in total 19 are without the RIPK3 orthologue while 6 species would have RIPK3. These are all reptile species (turtles and lizards). The proposed chicken RIPK3 (https://www.ncbi.nlm.nih.gov/gene/415708) is a gene that has been wrongly annotated as RIPK3 of which the RefSeq sequences on the gene page are annotated as RIPK2 (https://www.ncbi.nlm.nih.gov/protein/XP_004944206.1). The gene is flanked by OSGIN1 and is in fact a paralog of RIPK2. Also in e.g. zebra finch this RIPK2-like gene (LOC115496886: https://www.ncbi.nlm.nih.gov/gene/115496886) is flanked by OSGIN1. Note that the true RIPK2 gene is flanked by OSGIN2 in both the chicken, zebra finch and human genome. Although RIPK5/ANKK1 is absent in the current genome assemblies of some reptiles, some more recently sequenced reptile species do have an ANKK1 orthologue. To confirm this, one can look at the genomic region containing the ANKK1 flanking genes TTC12 and DRD2. Noteworthy, this region in the anole lizard (*Anolis carolinensis*) genome does not contain an ANKK1 gene although that region in the current assembly contains gaps. It is interesting to note that the appearance of RIPK4, which was shown to have an important role in skin barrier formation [19, 20], coincides with the appearance of tight junctions and claudins in vertebrates [21–23], where the epithelial barriers became more diverse and sophisticated. Tight junctions are specific to vertebrates, however homologous structures with similar functions can also be found in invertebrates, consisting of known tight junction components (e.g. claudin-like proteins), but differing in localization and components [23].

All RIPKs show homology in their kinase domain, but have different C-terminal functional domains (Fig. 1A). Domain switching experiments revealed that the kinase domains of RIPK2 and RIPK4 are functionally similar in regard of their ability to induce NF-κB activation, however the RIPK2 kinase domain is unique in its ability to act downstream of NOD2 signaling [24]. Evolutionary seen, RIPK2 and RIPK4 are closer to each other than RIPK1 or RIPK3, since they diverged from a common ancestor later than divergence to RIPK1 and RIK3 (Fig. 1b). RIPK5 (ANKK1), a RIPK4 paralog, is structurally very similar to RIPK4, but there is little knowledge on which signaling pathways involve RIPK5/ANKK1 and polymorphisms in the RIPK5 gene are mainly linked to neuropsychiatric disorders such as addiction [25]. In addition, there were no skin abnormalities reported in RIPK5/ANKK1-deficient mice [26].

## Functions of RIPKs in the skin

Apart from RIPK5 (ANKK1), which is often linked to neurological disorders, all RIPKs function as crucial integrators of cellular stress signals such as inflammation, cytokines, DNA damage, pathogen infections or differentiation. RIPKs are thus implicated in many processes. RIPK2-deficient mice do not display a skin phenotype. The NOD2–RIPK2 signaling axis has however been shown to play a role in inflammatory diseases such as early-onset sarcoidosis (EOS), which involves a triad of skin, joint and eye defects [27]. Apart from one report implicating RIPK2 as a player in the regulation of keratinocyte proliferation and wound re-epithelialization there is no other evidence for a role of RIPK2 in keratinocyte proliferation or differentiation, but it rather plays a role in inflammatory signaling also affecting the skin [27–30]. RIPK2 seems mainly involved in keratinocyte responses to intracellular bacterial pathogens [31] and/or bacterial cell wall components [32]. In oral keratinocytes and squamous cell carcinoma (SCC) cell lines, the latter can induce RIPK2-dependent PD-L1 expression, a ligand involved in cell-mediated immune responses that can induce an immune-evasive microenvironment and prevent T-cell mediated destruction of cancer cells, suggesting that RIPK2 could play a role in preventing immune reaction to pathogens and/or to SCC [32]. RIPK1 and RIPK3 control and execute necroptosis in keratinocytes, as in other cell types, while RIPK4 controls proliferation and differentiation of keratinocytes and thereby can act as a tumor suppressor in skin. Therefore, we will further zoom in on the function of RIPK1 and -3 in keratinocyte cell death and the role of RIPK4 in epidermal differentiation and tumor suppression.

### RIPK1 and RIPK3 in TNF signaling

RIP1K contains an N-terminal serine/threonine kinase domain, an intermediate domain and a C-terminal death domain (Fig. 1A). RIPK1 was discovered in a genetic screen for Fas-interacting proteins and was found to interact with the intracellular domains of Fas and p55 tumor necrosis factor receptor (TNFR1) through its C-terminal death domain (DD) [33]. The intermediate domain harbors a receptor-interacting protein homotypic interaction motif (RHIM) that allows interaction with other RHIM-containing proteins such as RIPK3. RIPK3 has an N-terminal kinase domain, a RHIM domain and a unique C-terminus with no sequence similarities to any known protein domain (Fig. 1A) [3, 34, 35].

**Fig. 2:**
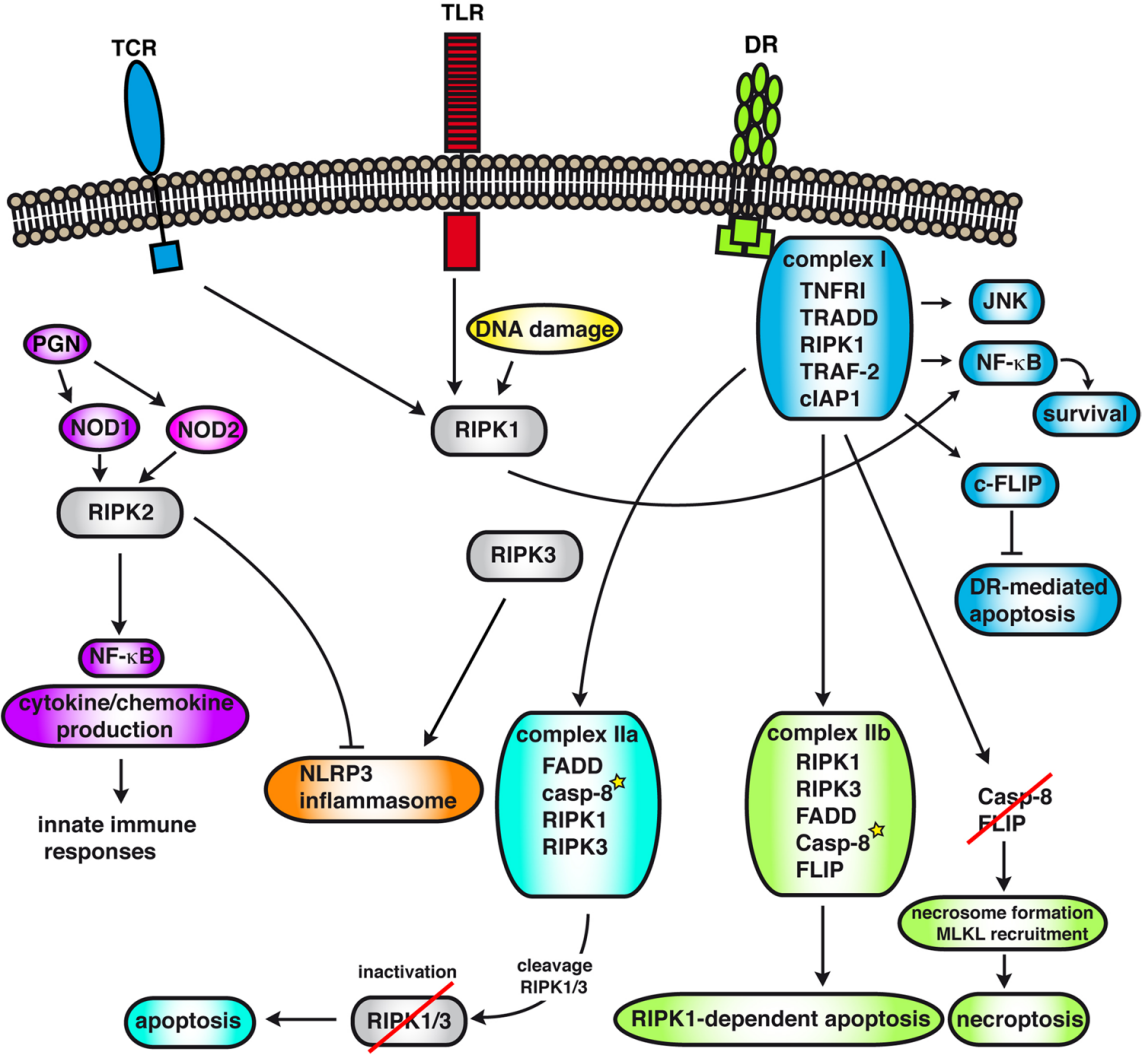
RIPK1, 2 and 3-mediated signaling pathways. Death receptor (DR) activation results in the formation of a membrane-bound complex I, which can activate JNK and other MAPKs and NF-κB resulting in survival signals. After internalization it is believed that complex I dissociates from the receptor and can lead to different secondary complexes depending on the cellular context (complex IIa/b or the necrosome). In complex IIa, RIPK1 and 3 are inactivated through cleavage by activated caspase-8, which results in the activation of the apoptotic signaling cascade and apoptosis. For some death receptors (Fas, TRAIL) FADD and caspase-8 can be recruited to the receptor complex resulting in caspase-8 activation at the receptor complex. In both cases caspase-8 activation is RIPK1-independent. Complex II signaling can also result in RIPK1-dependent necrosome formation and necroptosis in absence of caspase-8 activity or RIPK1-dependent apoptosis when caspase-8 is active. RIPK1 is also involved in inflammatory signaling downstream of TLR and TCR. RIPK2 is required for integrating signals from NOD1/2, which are cytosolic receptors for bacterial peptidoglycan (PGN) derivatives. RIPK2/NOD signaling results in NF-κB activation, cytokine and chemokine production and is important during innate immune responses. RIPK2/NOD2 signaling was shown to inhibit the NLRP3 inflammasome, while RIPK3 can promote NLRP3 activation*.* *TCR* T-cell receptor, *TLR* toll-like receptor, *DR* death receptor

Apart from its role in inducing cell death, RIPK1 can also activate NF-κB downstream from death receptors, TLR3 and TLR4 [36], and T cell antigen receptor [37]. RIPK1-deficient mice present with extensive apoptosis in both, adipose and lymphoid tissues, leading to death at 1–3 days of age, indicating an important role of RIPK1 in cell survival in vivo [38]. Upon ligand (TNF) binding to TNFR1 or other death receptors (DR), two sequential, RIPK1-containing complexes are formed (complex I and II). Upon TNFR1 receptor engagement, the receptor-bound complex I is formed by recruitment of specific adaptor proteins (Fig. 2). Complex I consists of TNFR1, TRADD, RIPK1, and the E3 ubiquitin ligases TRAF-2 (TNF-receptor associated factor 2) and cIAP1 (cellular inhibitor of apoptosis). Recruitment of death domain-containing adaptor protein TRADD (TNFR associated death domain) promotes proinflammatory signaling [39, 40]. Complex I formation induces rapid polyubiquitination of RIPK1, recruitment of TAK1 (TGFβ-activated kinase 1), TAB1/2 (TAK-1-binding proteins) and IKK (IκB kinase) complexes and NF-κB activation, independent of RIPK1 kinase activity [3, 41–44]. Complex I signaling also leads to the expression of antiapoptotic proteins such as c-FLIP (FADD-like IL-1β-converting enzyme inhibitory protein), which potently inhibits death-receptor mediated apoptosis [3, 45–48]. RIPK1-deficient mouse embryonic fibroblasts (MEFs) are highly sensitive to TNF-induced cell death [38]. Some studies claim an important role for RIPK1 in TNF-induced NF-κB in MEFs [38], while others report only a partial impact of RIPK1 deletion on the expression of NF-κB target genes [49], suggesting the requirement for RIPK1 in TNF-induced NF-κB activation may depend on the cellular conditions, cell type or cell line. Shortly after TNF stimulation, RIPK1 and TRAF2 are deubiquinated by CYLD (cylindromatosis), a cysteine protease reported clipping off Lys63-linked and linear ubiquitin chains [50], thereby disrupting their scaffolding function, which is required for TAK1/IKK recruitment. Of note, depending on the cell type, RIPK1 is not even required for TNF-induced NF-κB activation, which is the case in keratinocytes [51, 52]. The fact that RIPK1 is dispensable for TNF-induced NF-κB activation in some cell types but not in others, may be due to the redundant role of RIPK1 and TRADD in TNFR1-dependent NF-κB activation [53, 54]. Therefore, the levels of RIPK1 and TRADD in the different cell types may define the impact of RIPK1 deletion on NF-κB activation. Depending on the conditions, RIPK3 was shown to be able to activate or inhibit NF-κB activation [35, 55–58]. Since RIPK3-deficient MEFs and macrophages (BMDMs) respond normally to TNF- or LPS-induced NF-κB activation [59], the role of RIPK1 and RIPK3 may not be crucial for NF-κB activation by certain triggers.

TNFRI engagement can also lead to the formation of secondary cytoplasmic death complexes depending on the cellular context, known as complex IIa/IIb and necrosome (Fig. 2). They are formed after dissociation and internalization of complex I and its components [53, 60]. The current models suggest that in complex IIa (TRADD, FADD, caspase-8, RIPK1, RIPK3), RIPK1 and 3 are inactivated by caspase-8-dependent cleavage, which results in the activation of the proteolytic caspase cascade and apoptotic cell death. RIPK1/3 cleavage by caspase-8 is critical for preventing extensive necroptosis during embryonic development [60]. Complex IIb (RIPK1, RIPK3, FADD, caspase-8, FLIP) signaling can result in RIPK1-mediated kinase-dependent caspase-8 activation eventually leading to apoptosis. In absence of caspase-8 activity (e.g. during viral infections), active RIPK1 recruits RIPK3 leading to its activation. RIPK3 can then recruit and activate mixed lineage kinase domain-like (MLKL) by transphosphorylation, resulting in the pore-forming conformation of MLKL that permeabilizes the cellular membrane leading to necroptosis [61–65]. Necrosome formation, regulation and signaling have been extensively reviewed elsewhere and are not discussed here [18, 65–69].

### RIPK1 and RIPK3 in keratinocyte cell death

RIPK1 kinase activity can lead to caspase-8 activation resulting in keratinocyte apoptosis and skin inflammation and in certain mouse models RIPK1 kinase-dead (D138N/D138N) knock-in mice (RIPK1^kd/kd^) are protected against keratinocyte cell death and skin inflammation. This can occur when NF-κB signaling is hampered in keratinocytes, such as in IKK2^EKO^ mice [70], combined RelA^EKO^c-Rel^EKO^ mice lacking two essential NF-κB subunits [70], Sharpin^cpdm/cpdm^-deficient mice [71], mice lacking cIAP1/cIAP2 in the epidermis [72], or in mice lacking NF-κB subunits [73]. cIAPs (inhibitor of apoptosis) and Sharpin are important mediators of NF-κB signaling. Whether the spontaneous skin inflammation observed in these mice strains is due to the fact that IKK2 can dampen RIPK1 activity [74], explaining the huge sensitization towards TNF-induced RIPK1-dependent cell death, or to the fact that the NF-κB transcription factor is no longer activated in keratinocytes, causing RIPK1-dependent TNF-induced cell death [73, 75], or both, is currently unclear.

RIPK3 and MLKL are dispensable for skin development and homeostasis because mice deficient in these genes do not show a spontaneous skin phenotype. In contrast, keratinocyte-specific ablation of FADD, caspase-8 or RIPK1 leads to severe skin inflammation and mortality [51, 76, 77], illustrating their importance for epidermal homeostasis. RIPK3 is negatively regulated by caspase-8 and FADD, which induce RIPK3, RIPK1 and CYLD cleavage and thereby inhibit necroptosis [78]. Histological analysis suggested that the absence of FADD, caspase-8 or RIPK1 in keratinocytes sensitizes them for necroptotic cell death in vivo [51, 76], indicating that RIPK1 is dispensable for necroptosis. This observation was genetically confirmed by intercrossing these mutant mice strains with RIPK3- or MLKL-deficient mice, which completely rescued the skin inflammation phenotype [51, 79–82]. The necroptosis-inducing ability of RIPK3 can be dampened by the deubiquitinating function of A20 [83]. Because patients carrying mutations in the A20 gene are genetically predisposed to develop psoriatic disease (reviewed in [84]), it would be interesting to evaluate the contribution of RIPK1 or 3 in the development of skin inflammation in these patients. Recently it was shown that a novel RIPK1 inhibitor could ameliorate skin inflammation in the IMQ-induced psoriasiform mouse model [85].

Interestingly, RIPK1^kd/kd^ knock-in mice do not show a spontaneous phenotype [51, 52, 71] indicating that RIPK1 can have a kinase-independent platform function needed to protect cells against sensitization to cell death and a kinase-dependent function leading to cell death.

While RIPK1 has recently been shown to play an essential role in preventing epithelial cell apoptosis and necroptosis [51, 52], RIPK3, although expressed in keratinocytes, is dispensable for normal epidermal development. Keratinocyte-specific ablation of RIPK1 in mice results in severe inflammatory skin lesions that are characterized by the occurrence of dying keratinocytes, epidermal thickening and increased expression of inflammatory cytokines and chemokines. Skin inflammation in RIPK1^EKO^ mice can be rescued by RIPK3 or MLKL deficiency, both proteins crucial for necroptosis induction, indicating that inflammation in RIPK1^EKO^ is driven by RIPK3-MLKL-dependent keratinocyte necroptosis [51, 80, 82]. RIPK1 restrains RIPK3-mediated necroptosis through a kinase-independent scaffolding function [51]. One important question was which trigger ignites RIPK3-dependent keratinocyte necrosis in the absence of RIPK1. RIPK1^EKO^/TNFR1^−/−^ double knockout mice still develop patchy inflammatory skin lesions [51], but at a later stage than RIPK1^EKO^ mice (7–8 weeks compared to 2–3 weeks in RIPK1^EKO^ mice), suggesting involvement of additional triggers. Therefore, it was suggested that the RIPK1 RHIM prevents ZBP1/DAI, a RHIM domain containing Z-DNA or -RNA sensor, from binding and activating RIPK3 upstream of MLKL [86]. Indeed, skin inflammation and keratinocyte necroptosis in RIPK1^EKO^ mice can be delayed by ZBP1 deficiency or by crossing these mice to sensing-dead ZBP1 knockin mice [51, 80, 82, 86–88], indicating that not the mere absence of RIPK1 is sufficient to activate ZBP1-dependent RIPK3/MLKL-induced keratinocyte necroptosis but that endogenous nucleic acids are needed to ignite ZBP1-induced cell death under these conditions. Whether sensing of endogenous nucleic acids by ZBP1 is also implicated in other inflammatory diseases needs further investigation. Furthermore, skin lesions in RIPK1^EKO^ can be mildly ameliorated by keratinocyte-specific deletion of TRIF (TIR-domain-containing adapter-inducing interferon-β) [51]. This could fit with the role of ZBP1 in RIPK3-dependent necrosis in RIPK1^EKO^ mice since TRIF is a known inducer of interferons (IFNs) and ZBP1 is a target gene of IFN signaling (reviewed in [89]). Hence, one could expect that crossing RIPK1^EKO^ mice with KO mice with an abrogated IFN response, such as STAT1 KO mice [90], would also be largely rescued from skin inflammation.

Although it is evident from literature that FADD, caspase-8 and RIPK1 play an essential role in keratinocytes in mice to prevent unwanted cell death it is not clear whether there are pathological human conditions related to these findings. Patients carrying homozygous caspase-8 loss-of-function mutations have not been reported to develop skin lesions [91], probably due to the presence of the redundant caspase-10 in humans, which is lacking in mice. Patients with homozygous RIPK1 deficiency or mutations are rare and all patients suffer from growth failure, intestinal inflammation and T and B cell dysfunction, rather than from skin manifestations due to necroptotic keratinocyte cell death [92–98]. Why the phenotypes caused by RIPK1 deficiency differ between mice and men is currently not clear but may depend on differences in threshold levels of ZBP1, its ligand, or RIPK3, needed to drive necroptotic cell death in keratinocytes.

Recently, a role for RIPK3 in toxic epidermal necrolysis (TEN) has been described. RIPK3 is strongly upregulated in the epidermis of lesional skin in TEN patients and its expression coincides with increased MLKL phosphorylation in situ as well as with necroptotic signaling [99, 100]. In toxic epidermal necrolysis, keratinocytes die necrotically [101]. This cytotoxicity can be completely inhibited by Nec-1 and significantly decreased by knockdown of RIPK3 with small interfering RNA (siRNA) [102]. Whether ZBP1 could play a role in TEN has not been investigated. This argues for therapeutic testing of RIPK1 or RIPK3 inhibitors in certain skin inflammatory diseases. RIPK1 and MLKL inhibitors were shown to prevent skin inflammation in an imiquimod (IMQ)-induced psoriasiform skin inflammation model [103], suggesting a role for necroptosis in skin inflammation. It would be interesting to confirm these results in RIPK3- or MLKL-deficient mice. RIPK1 inhibitors are in phase IIa clinical trial for treating mild to moderate psoriasis, however further studies are needed to evaluate whether RIPK1 inhibitors have potential as new therapy for treating psoriasis [104]. Other studies however suggest that RIPK1 is rather downregulated in psoriatic skin [105]. Whether patients with a genetic defect in NF-κB activation, such as patients carrying hypomorphic IKKγ/NEMO mutations (lack of NF-κB activation), or reduced activity of the RIPK1 deubiquitinating enzyme A20/TNFAIP3 (more NF-κB activation), which both suffer from skin inflammation [67, 106], would benefit from treatment with RIPK inhibitors needs further investigation.

### RIPK4

**Fig. 3:**
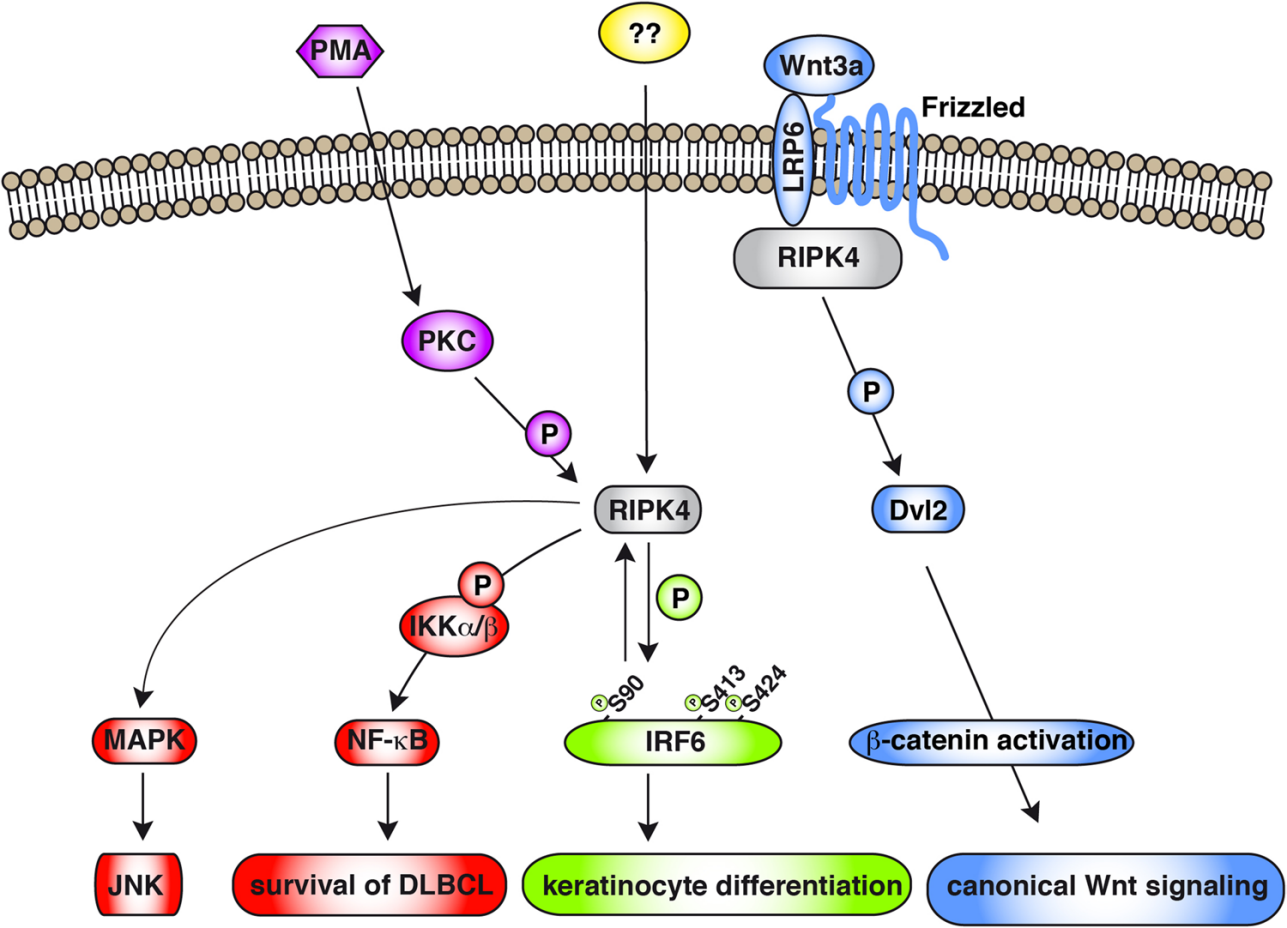
RIKP4-mediated signaling pathways. RIPK4 signaling can lead to the activation of NF-κB through interaction and phosphorylation of IKKα/β and is dependent on the presence of IKKβ, but not IKKγ (NEMO). RIPK4-induced NF-κB activation was shown to be essential for survival of diffuse large B-cell lymphoma (DLBCL). Other functional implications of RIPK4-dependent NF-κB activation are currently unknown. Upon Wnt3a stimulation, RIPK4 is recruited to LRP6 (co-receptor of Frizzled) and directly phosphorylates Dvl2, which leads to cytosolic β-catenin accumulation and results in activation of canonical Wnt signaling. Furthermore, RIPK4 signaling can lead to activation of MAPK and JNK. RIPK4 can interact with PKCs and can be phosphorylated by them. PKCs can be activated by PMA/phorbol esters. However, the physiological trigger activating PKC-mediated RIPK4 activation remains enigmatic. PMA-stimulation results in RIPK4-dependent IRF6 phosphorylation and activation, leading to the upregulation of IRF6 target genes, which promote keratinocyte differentiation. Some experiments indicate that RIPK4 and IRF6 can regulate each others expression

Human RIPK4 has originally been identified as protein kinase C (PKC)-δ-interacting protein (DIK) [107]. The mouse orthologue of RIPK4, also called PKK (protein kinase C-associated kinase), has been shown to interact with PKC-βI [108]. RIPK4 consists of an N-terminal RIP-homologous kinase domain, followed by an intermediate domain and eleven C-terminal ankyrin repeats (Fig. 1A). RIPK4 has been shown to be an important mediator in NF-κB activation, Wnt signaling and keratinocyte differentiation (Fig. 3). Based on the number of genetic mouse models, it is clear that RIPK4 plays an important role in epidermal development and keratinocyte differentiation [19, 20, 109–113].

The X-ray crystal structure of murine RIPK4 and biochemical experiments show that RIPK4 needs to act as a dimer to be enzymatically active [114], and that it is structurally similar to the other RIPKs. The kinase domain of RIPK4 has been shown to be sufficient to activate NF-κB and JNK. RIPK4 is also capable of autophosphorylation, as occurring in other RIP kinases [34, 115]. Unlike RIPK1 and RIPK2, RIPK4-induced NF-κB activation is dependent on its kinase activity, which directly phosphorylates IKK1 and IKK2 [116], and on the presence of IKK2 but not of NEMO/IKK [117]. Furthermore, RIPK4 can be cleaved by caspases in its intermediate domain during apoptosis [34]. Although RIPK4 overexpression in keratinocytes can induce the expression of several NF-κB-regulated inflammatory cytokines [118] the functional physiological implications of RIPK4-induced NF-κB and JNK activation or caspase-8 mediated cleavage are currently not clear. PMA-stimulated keratinocytes produce these NF-κB-regulated cytokines in a RIPK4-dependent way [118], suggesting that RIPK4-mediated cytokine production could play a role in skin inflammatory reactions. Of course, PMA is a general activator of classical and novel PKCs and it is difficult to judge which physiological stimulus matches with PMA stimulation of cells. RIPK4 mRNA expression is downregulated in the proliferating keratinocytes at the wound edge of incisional wounds in mice [119], which would fit with the observation that in vivo RIPK4 deletion in keratinocytes leads to a hyperproliferative phenotype (refs. in Table 1). In addition, it was shown in that in vitro RIPK4 knockdown in the HaCaT keratinocyte cell line leads to increased proliferation [120] and in vivo deletion of RIPK4 in mice results in a hyperproliferative phenotype (see refs in Table 1). In contrast, the hyperproliferative epidermis in psoriatic patients shows increased levels of RIPK4, possibly derived from IL-17-stimulated keratinocytes [121]. To clarify the potential role of RIPK4 in wound healing or psoriatic disease, it may be interesting to investigate the response of RIPK4^iEKO^ mice in wound healing models or in skin inflammation models such as imiquimod treatment. However, one should consider that these mice have a reduced skin barrier that may hamper the interpretation of the results when such mice would be topically treated with inflammatory agents.

**Table 1:** Mouse gene knockout models with similar phenotypes as RIPK4-deficient mice

Phenotype	Gene knockout model												
	RIPK4^−/−^ (*1)	RIPK4^EKO^	RIPK4^iEKO^ (*2)	IRF6 (*3)	IRF6^EKO^	IKK1^−/−^	IKK1^EKO^	IKK1^iEKO^ (*2)	GRHL3^−/−^	GRHL3^EKO^	KDF1^−/−^ (*4)	Er/Er (*5)	Stratifin^−/−^ (14–3-3σ)
Die at birth	**+**	**+ **or − (*6)	**−**	**+**	**+**	**+**	** + **or **− **(*6)	**−**	**+**	**−**	**+**	**+**	**−**
Fusion of external orifices, oral cavity and esophagus	**+**	**−**	**−**	**+**	**−**	**+**	**−**	**−**	NR	**−**	**+**	**+**	**−**
Aberrant adhesion of the periderm	**+**	**−**	**−**	**+**	**−**	**+**	**−**	**−**	**+**	**−**	**+**	**+**	**−**
syndactyly	**+**	**−**	**−**	NR	**−**	**+**	**−**	**−**	**+**	**−**	**−**	**+**	**−**
Shorter limbs and tail	**+**	**−**	**−**	**+**	**−**	**+**	**−**	**−**	**−**	**−**	**+**	**+**	**−**
Skin barrier defect	**+**	**+**	**+**	**+**	**+**	**+**	**+**	NR	**+**	**−**	**+**	NR	**−**
Hyperproliferative epidermis	**+**	**+**	**+**	**+**	**+**	**+**	**+**	**+**	**+**	**+**	**+ **(*7)	**+**	**−**
Failure of terminal keratinocyte differentiation	**+**	**+**	**+**	**+**	**+**	**+**	**+**	NR	**+**	**+ **(*8)	**+**	**+**	**−**
Underdeveloped hair follicles	**+**	NR	NR (*9)	**+**	NR	**+**	NR	NR	NR	**−**	NR	**+**	**−** (*10)
Spontaneous skin tumors	NA	**+**	**+**	NA	NA	NA	NA	**+**	NA	**−**	NA	NA	**−**
Sensitized to DMBA/PMA-induced skin carcinogenesis	NA	**+**	**+**	NA	NA	**+ **(*11)	NA	NR	NA	**−**	NA	NA	**+**
Delayed embryonal wound healing or defective repair of epidermal injury in adults	NR	NR	NA	**+**	NR	NR	NR	NA	**+**	**+**	NR	NR	NR
References	[19, 20, 110, 112]	[20, 109, 138]	[20, 109]	[112, 147, 184]	[112]	[185–189]	[187, 190]	[187]	[191–196]	[191]	[155, 197]	[198–200]	[201]

Comparison of the major phenotypic characteristics of several mouse gene knockout or knockin models displaying similar phenotypes as RIPK4-deficient mice. The similarity in phenotypes may indicate several of these genes act in common signaling pathways. For some of these, this has already been proven, e.g. RIPK4 and IRF6, KDF1 and IKK1 (see text and table for references). *1: display patchy stretches of cornification; RIPK4^D161N/D161N^ kinase-dead knockin mice show the same phenotype; *2: also show increased sensitivity to constitutive active PI3K-induced skin carcinogenesis; *3: IRF6^R84C/R84C^ knockin mice carrying an IRF6 mutation found in Van der Woude syndrome patients, an ectodermal dysplasia syndrome, show a similar phenotype; *4: the skin phenotype is largely rescued by overexpression of IKK1; *5: this mutant mice strain carries a frameshift mutation in stratifin thereby expressing a C-terminal truncated form of stratifin that probably has altered activity since the stratafin-deficient mice display a much milder phenotype; *6: die at birth due to a defective skin barrier or survive, depending on the K14Cre line used; *7: hyperproliferative epidermis phenotype is p63-dependent; *8: the impairment of keratinocyte terminal differentiation is normalized at an age of about 2 months; *9: show patchy hair loss about 4–6 weeks after gene deletion; *10: display disorganized fur (due to a hair follicle defect?); *11: homozygous KO mice are not viable, but heterozygous IKK1^+/−^ mice are sensitized to DMBA/PMA-induced skin carcinogenesis

*NA* not applicable, *NR* not reported

RIPK4 can interact with PKCs and kinase-dead RIPK4 mutants can inhibit PMA/Ca2 + -ionophore-, but not TNF-, IL1- or NOD1-induced NF-κB activation, placing RIPK4 downstream of PKC [117, 122]. PMA is a potent activator of conventional and novel PKCs and a known inducer of keratinocyte differentiation [123, 124]. Recently it has been shown that PKCδ, -ε, and -η can induce a RIPK4 phosphorylation band shift upon overexpression with kinase dead RIPK4-K51R, suggesting that these PKC family members could be involved in PMA-induced RIPK4 activation [115]. RIPK4 is constitutively activated in in vitro proliferating keratinocytes, reflected by the presence of a phosphorylated RIPK4 protein band in addition to nonphosphorylated RIPK4 in western blot experiments [115]. It would be interesting to investigate which PKC isoforms are involved in this constitutive activation. The levels of activated RIPK4 are kept low in keratinocytes by SCFβ-TrCP-dependent proteasomal RIPK4 degradation to prevent actin cytoskeletal reorganizations that lead to increased keratinocyte motility [115]. The biological rationale to limit keratinocyte motility in homeostatic conditions could be that this is important to guarantee strong interaction between the keratinocytes sealing the body.

RIPK4 activity can also transmit Wnt signaling in several non-keratinocyte cell lines leading to stabilization of β-catenin eventually inducing Wnt target gene expression [125–127]. However, in bladder carcinoma cells RIPK4 deletion results in higher levels of β-catenin and RIPK4 overexpression rather correlated with a more mesenchymal phenotype of the cells [128]. Upon Wnt3a treatment of HEK293T cells, RIPK4 is recruited to LRP6, a co-receptor of frizzled (Fz) receptor, and phosphorylates the adaptor protein Dishevelled 2 (DVL2) at S298 and S480, which initiates canonical Wnt signaling. Wnt signaling has a crucial function during skin development and is involved from the earliest stages of skin development to controlling the functions of differentiated keratinocytes [129, 130]. Wnt ligands as well as frizzled receptors are expressed in the skin at E14.5 [131, 132]. Wnt3 and DVL2 were shown to be important for hair shaft formation [133] and mice with epidermis-specific β-catenin deletion do not induce the first postnatal anagen phase [134]. Due to its broad role in embryogenesis, knockout mice for Wnt signaling pathway components often have severe malformations (e.g. neural tube closure defects, cardiovascular defects), however, these phenotypes differ substantially from those observed in RIPK4 knockout mice [19, 110, 126, 135]. RIPK4-induced DVL2 phosphorylation and Wnt signaling may thus only control certain functions of Wnt in a spatiotemporal manner depending on the cellular context, such as tight junction protein expression in the epidermis [136, 137]. We conclude that the role of RIPK4 in Wnt signaling in different cell types, including keratinocytes, is currently not clear and should be further studied.

RIPK4 is widely expressed in most tissues. During embryogenesis, it is strongly expressed throughout the gastrointestinal tract, in particular the luminal tissues of the esophagus, stomach, duodenum and intestine as well as the skin from 14.5 dpc on [108]. Full RIPK4 knockout mice (RIPK4^−/−^) or kinase-dead knockin mice (RIPK4^KD−KI^) have severe ectoderm-derived organ abnormalities, cleft palate and E-cadherin-dependent epithelial fusions in the oral cavity and esophagus, leading to perinatal death by suffocation and show epidermal hyperproliferation and abnormal expression of keratinocyte differentiation markers [19, 20, 110, 112]. In addition, the RIPK4-deficient skin is covered by a thick proliferating layer of periderm cells, identified by expression of keratin 17 and keratin 6. Underneath this periderm layer, internal cornified patches are present. The absence of a continuous cornified layer however leads to a defective skin barrier.

Despite evidence for cornification in vivo, primary keratinocytes isolated from RIPK4-deficient mice fail to differentiate in vitro [19]. Depending on the genetic line, RIPK4^EKO^ mice die shortly after birth due to excessive water loss [20], probably because of lack of tight junction protein claudin-1 localization at the cell membrane, resulting in tight junction leakiness. Although the outside-in skin barrier in RIPK4^EKO^ mice was largely intact at the trunk in contrast to the skin covering the head and the outer end of the extremities, the inside-out barrier was overall defective in these RIPK4^EKO^ mice. At the ultrastructural level these mice show delayed keratinization and stratum corneum maturation as well as altered lipid organization [20], suggesting RIPK4 is indispensable during embryonic development for the formation of a functional inside-out epidermal barrier. Similar RIPK4^EKO^ mouse lines, using a different K14-Cre line or Cre-expressing lentiviral transduction to obtain RIPK4 ablation in the keratinocytes, survive but the mice are smaller in size and develop patchy hair loss and show a thickened epidermis [111, 138]. Lee and colleagues showed that the desmosomal protein plakophilin-1 (PKP1) is a target for RIPK4 phosphorylation [111]. Furthermore, these authors showed using grafting experiments that a phosphomimetic PKP1 mutant overexpressed in RIPK4-deficient keratinocytes could rescue the epidermal thickening of RIPK4-deficient epidermis. The authors suggested a model in which RIPK4-dependent PKP1 phosphorylation leads to association with SHOC2, a Ras–Raf scaffolding protein, thereby preventing overactivation of the Erk signaling pathway [111]. Mice with tamoxifen-induced keratinocyte-specific RIPK4 deletion during adult life (RIPK4^iEKO^) remain viable, however show a mild inside-out barrier defect and epidermal hyperproliferation, and also develop patchy hair loss [20, 109, 111], indicating that depending on the timing of RIPK4 deletion different phenotypes may develop.

Biochemical, cellular and genetic experiments suggest that RIPK4 activates the IRF6 transcription factor by phosphorylation at Serine 90, 413 and 424 and that its transient translocation to the nucleus eventually leads to upregulation of genes involved in keratinocyte differentiation and epidermal barrier formation such as involucrin (IVL), Patatin Like Phospholipase Domain Containing 1 (PNPLA1), Lipase G (LipG), desmocollin 1 (DSC1), occludin (OCLN), Elongation of Very Long Chain Fatty Acids Protein 4 (ELOVL4) and Grainyhead-like 3 (GRHL3) [112, 138, 139]. Keratinocyte-specific deletion of RIPK4 or IRF6 during embryogenesis or IRF6^S413A/S424A^ knockin mice show similar skin phenotypes and mice die shortly after birth due to severe skin barrier loss. RNA-seq data indicate that gene expression changes in embryonic skin of RIPK4 kinase-dead knockin mice were also observed in IRF6^−/−^ mice [20, 112]. Furthermore, it was suggested that PMA stimulation of human oral OKF6 keratinocytes results in RIPK4 activation (Fig. 3), which activates IRF6, resulting in expression of the IRF6 target GRHL3 eventually leading to ELF3 (ETS family transcription factor E74-like factor 3) expression. Downstream targets of GRHL3 and ELF3 include transglutaminase-1 and SPRR1 (small proline-rich protein 1), both important during cornified envelope formation, confirming the role of RIPK4 in regulating cornification [140]. It is thus believed that RIPK4 and IRF6 function in the same PKC-dependent signaling pathway to promote keratinocyte differentiation. IRF6 has also been reported to act upstream of RIPK4 since IRF6 dysfunction-induced developmental defects in Xenopus can be rescued by ectopic expression of wild-type RIPK4 and IRF6 overexpression can activate a RIPK4 promotor reporter construct [19]. In addition, overexpression of a dominant-negative variant of IRF6 inhibited RIPK4 expression (Supplementary Table in ref [141]). These findings suggest there may exist an oscillating regulatory loop between RIPK4 and IRF6.

Homozygous mutations in RIPK4 cause the autosomal recessive form of Popliteal Pterygium Syndrome (PPS), the Bartsocas Papas syndrome (BPS). BPS is characterized by multiple popliteal pterygia, ankyloblephora, filiform bands between the jaws, cleft lip and/or palate, and syndactyly [142]. PPS has also been associated with mutations in IKK1 and the transcription factor IRF6 [142–145]. In addition, inactivating mutations of RIPK4 result in dysfunctioning IRF6 by prevention of its transactivator function and nuclear translocation, which contributes to the phenotypes observed in patients with PPS [19, 125, 139, 146–150]. In the past few years ten BPS-associated RIPK4 mutations have been described, of which three lead to a premature stop codon and all others result in amino acid substitutions. The mutations mainly occur in the kinase domain, however two mutations have been described in the ankyrin domain of RIPK4, [142, 143, 151–154]. The BPS-related mutations in the kinase domain were indeed shown to be inactivating mutations [114, 143]. This suggests that RIPK4 activity requires a functional kinase domain but that mutations within its ankyrin domain may also influence its activity through a yet unknown mechanism. The phenotypes of RIPK4, IRF6, 14-3-3σ (Sfn), KDF1, GRHL3 (Grainyhead-like 3) and IKK1 knock-out mice are similar and show phenotypes similar to PPS, including severe epithelial adhesions, craniofacial abnormalities in addition to abnormal keratinocyte differentiation, and syndactyly, making them ideal model systems to study PPS as well as clefting disorders (Table 1). These knockouts also show a hyperproliferative epidermis, suggesting that these genes control the balance between keratinocyte proliferation and differentiation. RIPK4 can transactive IRF6 and the epidermal defects observed in KDF1-deficient mice are largely IKK1-dependent [112, 139, 155]. Thus, there seems a large regulatory network present between RIPK4, IRF6, 14-3-3σ, KDF1, GRHL3 and IKK1 that ensures proper epidermal development and control proliferation and differentiation. In addition, these genes are also essential for periderm development. The periderm is a protective single layer covering the developing epidermis during embryogenesis in mice (from E10.5 till E16-17) and men (from gestational week 4 to 23) [150, 156]. The tight junctions at the apico-lateral borders of the peridermal cells prevent spreading of adhesion molecules, such as E-cadherin, to the apical surface, thereby preventing adhesion and fusion of epithelia in the developing embryo [150]. In RIPK4-deficient mice, it was genetically shown that aberrant E-cadherin localization at the apical site of the peridermal cells, probably due to a defect in the tight junctions, was indeed responsible for the observed epithelial fusions [19, 20]. Interestingly, all of the above-mentioned factors are regulated by or can regulate the transcription factor p63 [142, 157–162]. Further studying RIPK4, IRF6 and IKK1 knockout mice as well as their signaling pathways and interactions will help to understand the etiology of the different human syndromes and epidermal differentiation.

Several reports indicate a role for RIPK4 in skin cancer [163]. The suggested implication of RIPK4 in tumorigenesis ranges from tumor-suppressor to tumor-promoter to involvement in metastasis. Tumor growth of Wnt signaling-dependent embryonal carcinoma tumor cells was inhibited by knockdown of RIPK4 in xenograft experiments (Fig. 3), indicating an oncogenic role of RIPK4 in certain tumor types [125]. These findings are in agreement with the observation that increased RIPK4 expression or high RIPK4 copy number correlated with progression and poor prognosis in patients suffering from cervical squamous cell carcinoma, colorectal cancer, ovarian cancer or bladder urothelial carcinoma [164–166]. In addition, RIPK4 knockdown in diffuse large B-cell lymphoma (DLBCL) cells was shown to impair cell survival (Fig. 3), inhibit tumor growth in xenograft experiments as well as sensitize the cells to chemotherapeutic treatment, probably due to inhibition of RIPK4-induced NF-κB activity [167]. RIPK4 expression has also been shown to promote cancer cell migration and invasion in bladder carcinoma, cervical cancer and pancreatic cancer [168]. These reports suggest an oncogenic role of RIPK4 in cancer, although tail vein injections of lung adenocarcinoma cells overexpressing RIPK4 showed a reduced potential to invade and form tumors [169]. RIPK4 mutations have been identified in several epithelial tumor types, such as esophageal squamous cell carcinoma and squamous cell carcinoma [170–174]. Consulting The Cancer Genome Atlas (TCGA, https://www.cancer.gov/tcga) and the International Cancer Genome Consortium (ICGC, https://icgc.org) indicated that RIPK4 is most frequently mutated in cutaneous squamous cell carcinoma, with around 25% of the patients samples harbouring RIPK4 mutations. Melanoma is the cancer type in which RIPK4 is second mostly being mutated, with an average mutation rate of around 4% based on the curated data of the cBio Portal (https://www.cbioportal.org) and COSMIC database (https://cancer.sanger.ac.uk/cosmic). In cutaneous SCC and melanoma several nonsense, frameshift and missense mutations occur mainly in the kinase and ankyrin domains of RIPK4 [120, 173, 175]. For a nice graphical overview see https://proteinpaint.stjude.org/. Except for the nonsense or frameshift mutations that largely disrupt the kinase domain and will be inactivating mutations, it is difficult to predict which of the other somatic RIPK4 mutants reported in the cosmic database are real drivers of cancerogenesis or just neutral mutations with no or a minor effect on RIPK4 activity because when tumors evolve they often acquire many somatic mutations [176]. Therefore, it is worthwhile to test the activity status of the different cancer-associated RIPK4 mutants. Since 26% of the reported cSCC RIPK4 mutants are nonsense or frameshift mutations this suggest there is selection for inactivating RIPK4 mutations in cSCC (https://proteinpaint.stjude.org/).

Intriguingly, recent genetic evidence in mouse skin tumor models and cell lines supports a tumor suppressor function of RIPK4 in skin, lung and hepatocellular carcinoma. RIPK4^EKO^ mice were shown to spontaneously develop skin tumors and show increased tumor burden in DMBA/PMA-induced skin carcinogenesis models [109, 111, 138, 175, 177]. In mouse transgenic skin cancer models expressing an activating mutant of phosphatidylinositol 3-kinase alpha (PIK3CA^H1047R^) or HRas (HRas^G12V^), mutations known to drive human SCC, a RIPK4 kinase-dependent tumor suppressor role was shown [138, 175, 177]. In contrast, mice overexpressing RIPK4 in the epidermis do not show increased tumor formation when treated with DMBA/PMA [113], supporting the idea that in keratinocytes RIPK4 acts as a tumor suppressor rather than as a tumor promotor. Reduced RIPK4 levels in human keratinocytes and SCC cell lines were shown to increase cell proliferation and tumorigenesis in xenograft models and soft agar experiments [120, 178]. In line with these findings, RIPK4 overexpression in transformed hepatocytes results in almost complete inhibition of anchorage-independent growth and RIPK4 suppression increased the growth of hepatocellular carcinoma cells [179]. In a mouse model of lung adenocarcinoma, RIPK4 suppression resulted in cancer dedifferentiation and in human lung adenocarcinoma patient low RIPK4 mRNA levels were associated with poor tumor differentiation and reduced overall survival [169]. Furthermore, RIPK4 has been found to be expressed at higher levels in well-differentiated tongue squamous cell carcinomas (TSCC) compared to poorly differentiated TSCC [180] and its expression is decreased in SCC compared to normal surrounding skin [120]. Hence, down-regulation of RIPK4 expression or the occurrence of somatic mutations hampering RIPK4 activity may be two independent events promoting cell autonomous skin carcinogenesis. However, the spectacular acceleration of skin tumor formation in RIPK4^EKO^ mice may not be fully attributable to cell autonomous effects because these mice also show a skin barrier defect and overall mild inflammatory skin [20, 109, 111, 138], which may promote tumor formation in a noncell autonomous way. The fact that mere overexpression of Elongation of Very Long Chain Fatty Acids Protein 4 (ELOVL4), an enzyme important for the long-chain fatty acids elongation cycle which is involved in skin barrier formation and whose expression is controlled by the RIPK4-IRF6 signaling axis [138, 181], is sufficient to largely delay skin tumor formation in the Pik3ca^H1047R^;RIPK4^EKO^ cancer model may point to a combined effect of cell autonomous and loss of skin barrier effect in these mouse models [138].

The role of RIPK4 in cancer is thus double-sided and seems to depend on the cellular context. Therefore, RIPK4 acts in some cells as a tumor suppressor and in others as a tumor promotor. Similar to RIPK4, the role of NF-κB in different models of cancer as well as different tissues was shown to depend on the cellular context, as both, tumor suppressing and tumor promoting functions have been observed in different cancer models [182, 183]. It is currently not clear whether the oncogenic/tumor suppressing function of RIPK4 is associated with its ability to activate NF-κB. Whether the absence of RIPK4 or RIPK4 overactivity, depending on the tumor type, affects the response of the cancer cells to cell death-inducing chemotherapeutics has not been investigated. Such knowledge will be useful in determining the value of determining the status of RIPK4 activity as a prognostic marker.

## Concluding remarks

The ancestral RIPK-like protein probably originated in Protostomes (Lophotrochozoa) and can consequently also be found in nonvertebrate Deuterostomes. These RIPK-like proteins however only contain an N-terminal kinase domain and lack known additional protein–protein interaction domains at their C-terminus. From the vertebrates on, RIPK-like proteins acquired C-terminal protein–protein interaction domains such as a death domain, CARD domain, RHIM motif and ankyrin repeat domain. The actual RIPKs (RIPK1-5) thus appeared at dawn of the vertebrates, which have more sophisticated skin and require protective mechanisms for maintaining homeostasis and the skin barrier that protects them from influences from the environment. The regulatory functions of the RIPK family have proven essential for proper epidermal development and maintenance of homeostasis. RIPK1EKOs develop severe inflammatory skin lesions due to RIPK3-mediated necrosis. Under normal conditions, RIPK1 thus provides epithelial homeostasis and protection by restraining RIPK3-mediated keratinocyte necroptosis in the skin. Uncontrolled RIPK3 activity in the skin can lead to diseases such as toxic epidermal necrolysis by inducing massive keratinocyte necrosis. RIPK1 and 3 are thus important regulators of keratinocyte survival and regulatory mechanisms are essential to maintain epidermal homeostasis. Currently ongoing clinical trials will teach us whether RIPK1 inhibitors can be used in skin inflammatory diseases such as psoriasis. The fourth member of the RIPK family, RIPK4, plays a major role in the skin by regulating keratinocyte differentiation and cornification. Based on genetic evidence RIPK4 mainly acts as a tumor suppressor in the skin. This however does not exclude the possibility that RIPK4 inhibitors might be of use to treat other tumor types, such as DLBCL, pancreatic or bladder cancer. Taken together, the RIPK family is indispensable for proper epidermal development and their regulation is crucial to maintain homeostasis and prevent inflammatory diseases and cancer.

## Data Availability

Not applicable.

## References

[1] Manning G, Whyte DB, Martinez R, Hunter T, Sudarsanam S (2002). The protein kinase complement of the human genome. Science.

[2] Meylan E, Tschopp J (2005). The RIP kinases: crucial integrators of cellular stress. Trends Biochem Sci.

[3] Zhang D, Lin J, Han J (2010). Receptor-interacting protein (RIP) kinase family. Cell Mol Immunol.

[4] Peng J, Dong W, Chen Y, Mo R, Cheng JF, Hui CC, Mohandas N, Huang CH (2006). Dusty protein kinases: primary structure, gene evolution, tissue specific expression and unique features of the catalytic domain. Biochim Biophys Acta.

[5] Li K, Liu JW, Zhu ZC, Wang HT, Zu Y, Liu YJ, Yang YH, Xiong ZQ, Shen X, Chen R, Zheng J, Hu ZL (2014). DSTYK kinase domain ablation impaired the mice capabilities of learning and memory in water maze test. Int J Clin Exp Pathol.

[6] Sanna-Cherchi S, Sampogna RV, Papeta N, Burgess KE, Nees SN, Perry BJ, Choi M, Bodria M, Liu Y, Weng PL, Lozanovski VJ, Verbitsky M, Lugani F, Sterken R, Paragas N, Caridi G, Carrea A, Dagnino M, Materna-Kiryluk A, Santamaria G, Murtas C, Ristoska-Bojkovska N, Izzi C, Kacak N, Bianco B, Giberti S, Gigante M, Piaggio G, Gesualdo L, Vukic DK, Vukojevic K, Saraga-Babic M, Saraga M, Gucev Z, Allegri L, Latos-Bielenska A, Casu D, State M, Scolari F, Ravazzolo R, Kiryluk K, Al-Awqati Q, D'Agati VD, Drummond IA, Tasic V, Lifton RP, Ghiggeri GM, Gharavi AG (2013). Mutations in DSTYK and dominant urinary tract malformations. N Engl J Med.

[7] Lee JYW, Hsu CK, Michael M, Nanda A, Liu L, McMillan JR, Pourreyron C, Takeichi T, Tolar J, Reid E, Hayday T, Blumen SC, Abu-Mouch S, Straussberg R, Basel-Vanagaite L, Barhum Y, Zouabi Y, Al-Ajmi H, Huang HY, Lin TC, Akiyama M, Lee JYY, McLean WHI, Simpson MA, Parsons M, McGrath JA (2017). Large intragenic deletion in DSTYK underlies autosomal-recessive complicated spastic paraparesis, SPG23. Am J Hum Genet.

[8] Greggio E, Lewis PA, van der Brug MP, Ahmad R, Kaganovich A, Ding J, Beilina A, Baker AK, Cookson MR (2007). Mutations in LRRK2/dardarin associated with Parkinson disease are more toxic than equivalent mutations in the homologous kinase LRRK1. J Neurochem.

[9] Haugarvoll K, Toft M, Ross OA, White LR, Aasly JO, Farrer MJ (2007). Variants in the LRRK1 gene and susceptibility to Parkinson's disease in Norway. Neurosci Lett.

[10] Langston RG, Rudenko IN, Cookson MR (2016). The function of orthologues of the human Parkinson's disease gene LRRK2 across species: implications for disease modelling in preclinical research. Biochem J.

[11] Schulte EC, Ellwanger DC, Dihanich S, Manzoni C, Stangl K, Schormair B, Graf E, Eck S, Mollenhauer B, Haubenberger D, Pirker W, Zimprich A, Brucke T, Lichtner P, Peters A, Gieger C, Trenkwalder C, Mewes HW, Meitinger T, Lewis PA, Klunemann HH, Winkelmann J (2014). Rare variants in LRRK1 and Parkinson's disease. Neurogenetics.

[12] Zimprich A, Biskup S, Leitner P, Lichtner P, Farrer M, Lincoln S, Kachergus J, Hulihan M, Uitti RJ, Calne DB, Stoessl AJ, Pfeiffer RF, Patenge N, Carbajal IC, Vieregge P, Asmus F, Muller-Myhsok B, Dickson DW, Meitinger T, Strom TM, Wszolek ZK, Gasser T (2004). Mutations in LRRK2 cause autosomal-dominant parkinsonism with pleomorphic pathology. Neuron.

[13] Toyofuku T, Morimoto K, Sasawatari S, Kumanogoh A (2015). Leucine-rich repeat kinase 1 regulates autophagy through turning on TBC1D2-dependent Rab7 inactivation. Mol Cell Biol.

[14] Arranz AM, Delbroek L, Van Kolen K, Guimaraes MR, Mandemakers W, Daneels G, Matta S, Calafate S, Shaban H, Baatsen P, De Bock PJ, Gevaert K, Vanden Berghe P, Verstreken P, De Strooper B, Moechars D (2015). LRRK2 functions in synaptic vesicle endocytosis through a kinase-dependent mechanism. J Cell Sci.

[15] Bosgraaf L, Van Haastert PJ (2003). Roc, a Ras/GTPase domain in complex proteins. Biochim Biophys Acta.

[16] Civiero L, Bubacco L (2012). Human leucine-rich repeat kinase 1 and 2: intersecting or unrelated functions?. Biochem Soc Trans.

[17] Marin I, van Egmond WN, van Haastert PJ (2008). The Roco protein family: a functional perspective. FASEB J.

[18] Dondelinger Y, Hulpiau P, Saeys Y, Bertrand MJM, Vandenabeele P (2016). An evolutionary perspective on the necroptotic pathway. Trends Cell Biol.

[19] De Groote P, Tran HT, Fransen M, Tanghe G, Urwyler C, De Craene B, Leurs K, Gilbert B, Van Imschoot G, De Rycke R, Guerin CJ, Holland P, Berx G, Vandenabeele P, Lippens S, Vleminckx K, Declercq W (2015). A novel RIPK4-IRF6 connection is required to prevent epithelial fusions characteristic for popliteal pterygium syndromes. Cell Death Differ.

[20] Urwyler-Rosselet C, Tanghe G, Leurs K, Gilbert B, De Rycke R, De Bruyne M, Lippens S, Bartunkova S, De Groote P, Niessen C, Haftek M, Vandenabeele P, Declercq W (2018). Keratinocyte-specific ablation of RIPK4 allows epidermal cornification but impairs skin barrier formation. J Investig Dermatol.

[21] Baltzegar DA, Reading BJ, Brune ES, Borski RJ (2013). Phylogenetic revision of the claudin gene family. Mar Genom.

[22] Mukendi C, Dean N, Lala R, Smith J, Bronner ME, Nikitina NV (2016). Evolution of the vertebrate claudin gene family: insights from a basal vertebrate, the sea lamprey. Int J Dev Biol.

[23] Zihni C, Mills C, Matter K, Balda MS (2016). Tight junctions: from simple barriers to multifunctional molecular gates. Nat Rev Mol Cell Biol.

[24] Chirieleison SM, Kertesy SB, Abbott DW (2016). Synthetic biology reveals the uniqueness of the RIP kinase domain. J Immunol.

[25] Koeneke A, Ponce G, Troya-Balseca J, Palomo T, Hoenicka J (2020). Ankyrin repeat and kinase domain containing 1 gene, and addiction vulnerability. Int J Mol Sci.

[26] Powell DR, Revelli JP, Doree DD, DaCosta CM, Desai U, Shadoan MK, Rodriguez L, Mullens M, Yang QM, Ding ZM, Kirkpatrick LL, Vogel P, Zambrowicz B, Sands AT, Platt KA, Hansen GM, Brommage R (2021). High-throughput screening of mouse gene knockouts identifies established and novel high body fat phenotypes. Diabetes Metab Syndr Obes.

[27] Jun JC, Cominelli F, Abbott DW (2013). RIP2 activity in inflammatory disease and implications for novel therapeutics. J Leukoc Biol.

[28] Adams S, Valchanova RS, Munz B (2010). RIP2: a novel player in the regulation of keratinocyte proliferation and cutaneous wound repair?. Exp Cell Res.

[29] Marcinek P, Jha AN, Shinde V, Sundaramoorthy A, Rajkumar R, Suryadevara NC, Neela SK, van Tong H, Balachander V, Valluri VL, Thangaraj K, Velavan TP (2013). LRRK2 and RIPK2 variants in the NOD 2-mediated signaling pathway are associated with susceptibility to Mycobacterium leprae in Indian populations. PLoS One.

[30] Takeuchi M, Mizuki N, Meguro A, Ombrello MJ, Kirino Y, Satorius C, Le J, Blake M, Erer B, Kawagoe T, Ustek D, Tugal-Tutkun I, Seyahi E, Ozyazgan Y, Sousa I, Davatchi F, Francisco V, Shahram F, Abdollahi BS, Nadji A, Shafiee NM, Ghaderibarmi F, Ohno S, Ueda A, Ishigatsubo Y, Gadina M, Oliveira SA, Gul A, Kastner DL, Remmers EF (2017). Dense genotyping of immune-related loci implicates host responses to microbial exposure in Behcet's disease susceptibility. Nat Genet.

[31] Eng VV, Wemyss MA, Pearson JS (2021). The diverse roles of RIP kinases in host-pathogen interactions. Semin Cell Dev Biol.

[32] Groeger S, Denter F, Lochnit G, Schmitz ML, Meyle J (2020). Porphyromonas gingivalis cell wall components induce programmed death ligand 1 (PD-L1) expression on human oral carcinoma cells by a receptor-interacting protein kinase 2 (RIP2)-dependent mechanism. Infect Immun.

[33] Stanger BZ, Leder P, Lee TH, Kim E, Seed B (1995). RIP: a novel protein containing a death domain that interacts with Fas/APO-1 (CD95) in yeast and causes cell death. Cell.

[34] Meylan E, Martinon F, Thome M, Gschwendt M, Tschopp J (2002). RIP4 (DIK/PKK), a novel member of the RIP kinase family, activates NF-kappa B and is processed during apoptosis. EMBO Rep.

[35] Sun X, Lee J, Navas T, Baldwin DT, Stewart TA, Dixit VM (1999). RIP3, a novel apoptosis-inducing kinase. J Biol Chem.

[36] Cusson-Hermance N, Khurana S, Lee TH, Fitzgerald KA, Kelliher MA (2005). Rip1 mediates the Trif-dependent toll-like receptor 3- and 4-induced NF-kappaB activation but does not contribute to interferon regulatory factor 3 activation. J Biol Chem.

[37] Zhang J, Zhang H, Li J, Rosenberg S, Zhang EC, Zhou X, Qin F, Farabaugh M (2011). RIP1-mediated regulation of lymphocyte survival and death responses. Immunol Res.

[38] Kelliher MA, Grimm S, Ishida Y, Kuo F, Stanger BZ, Leder P (1998). The death domain kinase RIP mediates the TNF-induced NF-kappaB signal. Immunity.

[39] Grootjans S, Vanden Berghe T, Vandenabeele P (2017). Initiation and execution mechanisms of necroptosis: an overview. Cell Death Differ.

[40] Seo J, Kim MW, Bae KH, Lee SC, Song J, Lee EW (2019). The roles of ubiquitination in extrinsic cell death pathways and its implications for therapeutics. Biochem Pharmacol.

[41] Ea CK, Deng L, Xia ZP, Pineda G, Chen ZJ (2006). Activation of IKK by TNFalpha requires site-specific ubiquitination of RIP1 and polyubiquitin binding by NEMO. Mol Cell.

[42] Legler DF, Micheau O, Doucey MA, Tschopp J, Bron C (2003). Recruitment of TNF receptor 1 to lipid rafts is essential for TNFalpha-mediated NF-kappaB activation. Immunity.

[43] Li H, Kobayashi M, Blonska M, You Y, Lin X (2006). Ubiquitination of RIP is required for tumor necrosis factor alpha-induced NF-kappaB activation. J Biol Chem.

[44] Wertz IE, O'Rourke KM, Zhou H, Eby M, Aravind L, Seshagiri S, Wu P, Wiesmann C, Baker R, Boone DL, Ma A, Koonin EV, Dixit VM (2004). De-ubiquitination and ubiquitin ligase domains of A20 downregulate NF-kappaB signalling. Nature.

[45] Degterev A, Hitomi J, Germscheid M, Ch'en IL, Korkina O, Teng X, Abbott D, Cuny GD, Yuan C, Wagner G, Hedrick SM, Gerber SA, Lugovskoy A, Yuan J (2008). Identification of RIP1 kinase as a specific cellular target of necrostatins. Nat Chem Biol.

[46] Micheau O, Tschopp J (2003). Induction of TNF receptor I-mediated apoptosis via two sequential signaling complexes. Cell.

[47] Ofengeim D, Yuan J (2013). Regulation of RIP1 kinase signalling at the crossroads of inflammation and cell death. Nat Rev Mol Cell Biol.

[48] Zhang SQ, Kovalenko A, Cantarella G, Wallach D (2000). Recruitment of the IKK signalosome to the p55 TNF receptor: RIP and A20 bind to NEMO (IKKgamma) upon receptor stimulation. Immunity.

[49] Wong WW, Gentle IE, Nachbur U, Anderton H, Vaux DL, Silke J (2010). RIPK1 is not essential for TNFR1-induced activation of NF-kappaB. Cell Death Differ.

[50] Sato Y, Goto E, Shibata Y, Kubota Y, Yamagata A, Goto-Ito S, Kubota K, Inoue J, Takekawa M, Tokunaga F, Fukai S (2015). Structures of CYLD USP with Met1- or Lys63-linked diubiquitin reveal mechanisms for dual specificity. Nat Struct Mol Biol.

[51] Dannappel M, Vlantis K, Kumari S, Polykratis A, Kim C, Wachsmuth L, Eftychi C, Lin J, Corona T, Hermance N, Zelic M, Kirsch P, Basic M, Bleich A, Kelliher M, Pasparakis M (2014). RIPK1 maintains epithelial homeostasis by inhibiting apoptosis and necroptosis. Nature.

[52] Takahashi N, Vereecke L, Bertrand MJ, Duprez L, Berger SB, Divert T, Goncalves A, Sze M, Gilbert B, Kourula S, Goossens V, Lefebvre S, Gunther C, Becker C, Bertin J, Gough PJ, Declercq W, van Loo G, Vandenabeele P (2014). RIPK1 ensures intestinal homeostasis by protecting the epithelium against apoptosis. Nature.

[53] Fullsack S, Rosenthal A, Wajant H, Siegmund D (2019). Redundant and receptor-specific activities of TRADD, RIPK1 and FADD in death receptor signaling. Cell Death Dis.

[54] Feoktistova M, Makarov R, Yazdi AS, Panayotova-Dimitrova D (2021). RIPK1 and TRADD regulate TNF-induced signaling and ripoptosome formation. Int J Mol Sci.

[55] Kaiser WJ, Upton JW, Mocarski ES (2008). Receptor-interacting protein homotypic interaction motif-dependent control of NF-kappa B activation via the DNA-dependent activator of IFN regulatory factors. J Immunol.

[56] Kasof GM, Prosser JC, Liu D, Lorenzi MV, Gomes BC (2000). The RIP-like kinase, RIP3, induces apoptosis and NF-kappaB nuclear translocation and localizes to mitochondria. FEBS Lett.

[57] Meylan E, Burns K, Hofmann K, Blancheteau V, Martinon F, Kelliher M, Tschopp J (2004). RIP1 is an essential mediator of Toll-like receptor 3-induced NF-kappa B activation. Nat Immunol.

[58] Yu PW, Huang BC, Shen M, Quast J, Chan E, Xu X, Nolan GP, Payan DG, Luo Y (1999). Identification of RIP3, a RIP-like kinase that activates apoptosis and NFkappaB. Curr Biol.

[59] Newton K, Sun X, Dixit VM (2004). Kinase RIP3 is dispensable for normal NF-kappa Bs, signaling by the B-cell and T-cell receptors, tumor necrosis factor receptor 1, and Toll-like receptors 2 and 4. Mol Cell Biol.

[60] Kaiser WJ, Upton JW, Long AB, Livingston-Rosanoff D, Daley-Bauer LP, Hakem R, Caspary T, Mocarski ES (2011). RIP3 mediates the embryonic lethality of caspase-8-deficient mice. Nature.

[61] Feng S, Yang Y, Mei Y, Ma L, Zhu DE, Hoti N, Castanares M, Wu M (2007). Cleavage of RIP3 inactivates its caspase-independent apoptosis pathway by removal of kinase domain. Cell Signal.

[62] Li J, McQuade T, Siemer AB, Napetschnig J, Moriwaki K, Hsiao YS, Damko E, Moquin D, Walz T, McDermott A, Chan FK, Wu H (2012). The RIP1/RIP3 necrosome forms a functional amyloid signaling complex required for programmed necrosis. Cell.

[63] Moriwaki K, Chan FK (2013). RIP3: a molecular switch for necrosis and inflammation. Genes Dev.

[64] Rodriguez DA, Weinlich R, Brown S, Guy C, Fitzgerald P, Dillon CP, Oberst A, Quarato G, Low J, Cripps JG, Chen T, Green DR (2016). Characterization of RIPK3-mediated phosphorylation of the activation loop of MLKL during necroptosis. Cell Death Differ.

[65] Vanden Berghe T, Linkermann A, Jouan-Lanhouet S, Walczak H, Vandenabeele P (2014). Regulated necrosis: the expanding network of non-apoptotic cell death pathways. Nat Rev Mol Cell Biol.

[66] Cuny GD, Degterev A (2021). RIPK protein kinase family: atypical lives of typical kinases. Semin Cell Dev Biol.

[67] Kondylis V, Kumari S, Vlantis K, Pasparakis M (2017). The interplay of IKK, NF-kappaB and RIPK1 signaling in the regulation of cell death, tissue homeostasis and inflammation. Immunol Rev.

[68] Kondylis V, Pasparakis M (2019). RIP kinases in liver cell death, inflammation and cancer. Trends Mol Med.

[69] Shan B, Pan H, Najafov A, Yuan J (2018). Necroptosis in development and diseases. Genes Dev.

[70] Kumari S, Van TM, Preukschat D, Schuenke H, Basic M, Bleich A, Klein U, Pasparakis M (2021). NF-kappaB inhibition in keratinocytes causes RIPK1-mediated necroptosis and skin inflammation. Life Sci Alliance.

[71] Berger SB, Kasparcova V, Hoffman S, Swift B, Dare L, Schaeffer M, Capriotti C, Cook M, Finger J, Hughes-Earle A, Harris PA, Kaiser WJ, Mocarski ES, Bertin J, Gough PJ (2014). Cutting Edge: RIP1 kinase activity is dispensable for normal development but is a key regulator of inflammation in SHARPIN-deficient mice. J Immunol.

[72] Anderton H, Rickard JA, Varigos GA, Lalaoui N, Silke J (2017). Inhibitor of apoptosis proteins (IAPs) limit RIPK1-mediated skin inflammation. J Investig Dermatol.

[73] Grinberg-Bleyer Y, Dainichi T, Oh H, Heise N, Klein U, Schmid RM, Hayden MS, Ghosh S (2015). Cutting edge: NF-kappaB p65 and c-Rel control epidermal development and immune homeostasis in the skin. J Immunol.

[74] Dondelinger Y, Jouan-Lanhouet S, Divert T, Theatre E, Bertin J, Gough PJ, Giansanti P, Heck AJ, Dejardin E, Vandenabeele P, Bertrand MJ (2015). NF-kappaB-independent role of IKKalpha/IKKbeta in preventing RIPK1 kinase-dependent apoptotic and necroptotic cell death during TNF signaling. Mol Cell.

[75] Xu C, Wu X, Zhang X, Xie Q, Fan C, Zhang H (2018). Embryonic lethality and host immunity of RelA-deficient mice are mediated by both apoptosis and necroptosis. J Immunol.

[76] Bonnet MC, Preukschat D, Welz PS, van Loo G, Ermolaeva MA, Bloch W, Haase I, Pasparakis M (2011). The adaptor protein FADD protects epidermal keratinocytes from necroptosis in vivo and prevents skin inflammation. Immunity.

[77] Kovalenko A, Kim JC, Kang TB, Rajput A, Bogdanov K, Dittrich-Breiholz O, Kracht M, Brenner O, Wallach D (2009). Caspase-8 deficiency in epidermal keratinocytes triggers an inflammatory skin disease. J Exp Med.

[78] O'Donnell MA, Perez-Jimenez E, Oberst A, Ng A, Massoumi R, Xavier R, Green DR, Ting AT (2011). Caspase 8 inhibits programmed necrosis by processing CYLD. Nat Cell Biol.

[79] Alvarez-Diaz S, Dillon CP, Lalaoui N, Tanzer MC, Rodriguez DA, Lin A, Lebois M, Hakem R, Josefsson EC, O'Reilly LA, Silke J, Alexander WS, Green DR, Strasser A (2016). The pseudokinase MLKL and the kinase RIPK3 have distinct roles in autoimmune disease caused by loss of death-receptor-induced apoptosis. Immunity.

[80] Lin J, Kumari S, Kim C, Van TM, Wachsmuth L, Polykratis A, Pasparakis M (2016). RIPK1 counteracts ZBP1-mediated necroptosis to inhibit inflammation. Nature.

[81] Zhang X, Fan C, Zhang H, Zhao Q, Liu Y, Xu C, Xie Q, Wu X, Yu X, Zhang J, Zhang H (2016). MLKL and FADD are critical for suppressing progressive lymphoproliferative disease and activating the NLRP3 inflammasome. Cell Rep.

[82] Devos M, Tanghe G, Gilbert B, Dierick E, Verheirstraeten M, Nemegeer J, de Reuver R, Lefebvre S, De Munck J, Rehwinkel J, Vandenabeele P, Declercq W, Maelfait J (2020). Sensing of endogenous nucleic acids by ZBP1 induces keratinocyte necroptosis and skin inflammation. J Exp Med.

[83] Onizawa M, Oshima S, Schulze-Topphoff U, Oses-Prieto JA, Lu T, Tavares R, Prodhomme T, Duong B, Whang MI, Advincula R, Agelidis A, Barrera J, Wu H, Burlingame A, Malynn BA, Zamvil SS, Ma A (2015). The ubiquitin-modifying enzyme A20 restricts ubiquitination of the kinase RIPK3 and protects cells from necroptosis. Nat Immunol.

[84] Harden JL, Krueger JG, Bowcock AM (2015). The immunogenetics of psoriasis: a comprehensive review. J Autoimmun.

[85] Guo Y, Jin L, Dong L, Zhang M, Kuang Y, Chen X, Zhu W, Yin M (2023). NHWD-1062 ameliorates inflammation and proliferation by the RIPK1/NF-kappaB/TLR1 axis in psoriatic keratinocytes. Biomed Pharmacother.

[86] Newton K, Manning G (2016). Necroptosis and inflammation. Annu Rev Biochem.

[87] Jiao H, Wachsmuth L, Kumari S, Schwarzer R, Lin J, Eren RO, Fisher A, Lane R, Young GR, Kassiotis G, Kaiser WJ, Pasparakis M (2020). Z-nucleic-acid sensing triggers ZBP1-dependent necroptosis and inflammation. Nature.

[88] Kesavardhana S, Malireddi RKS, Burton AR, Porter SN, Vogel P, Pruett-Miller SM, Kanneganti TD (2020). The Zalpha2 domain of ZBP1 is a molecular switch regulating influenza-induced PANoptosis and perinatal lethality during development. J Biol Chem.

[89] Kuriakose T, Kanneganti TD (2018). ZBP1: innate sensor regulating cell death and inflammation. Trends Immunol.

[90] Meraz MA, White JM, Sheehan KC, Bach EA, Rodig SJ, Dighe AS, Kaplan DH, Riley JK, Greenlund AC, Campbell D, Carver-Moore K, DuBois RN, Clark R, Aguet M, Schreiber RD (1996). Targeted disruption of the Stat1 gene in mice reveals unexpected physiologic specificity in the JAK-STAT signaling pathway. Cell.

[91] Su HC, Lenardo MJ (2008). Genetic defects of apoptosis and primary immunodeficiency. Immunol Allergy Clin N Am.

[92] Cuchet-Lourenco D, Eletto D, Wu C, Plagnol V, Papapietro O, Curtis J, Ceron-Gutierrez L, Bacon CM, Hackett S, Alsaleem B, Maes M, Gaspar M, Alisaac A, Goss E, AlIdrissi E, Siegmund D, Wajant H, Kumararatne D, AlZahrani MS, Arkwright PD, Abinun M, Doffinger R, Nejentsev S (2018). Biallelic RIPK1 mutations in humans cause severe immunodeficiency, arthritis, and intestinal inflammation. Science.

[93] Li Y, Fuhrer M, Bahrami E, Socha P, Klaudel-Dreszler M, Bouzidi A, Liu Y, Lehle AS, Magg T, Hollizeck S, Rohlfs M, Conca R, Field M, Warner N, Mordechai S, Shteyer E, Turner D, Boukari R, Belbouab R, Walz C, Gaidt MM, Hornung V, Baumann B, Pannicke U, Al Idrissi E, Ali Alghamdi H, Sepulveda FE, Gil M, de Saint BG, Honig M, Koletzko S, Muise AM, Snapper SB, Schwarz K, Klein C, Kotlarz D (2019). Human RIPK1 deficiency causes combined immunodeficiency and inflammatory bowel diseases. Proc Natl Acad Sci USA.

[94] Uchiyama Y, Kim CA, Pastorino AC, Ceroni J, Lima PP, de Barros DM, Honjo RS, Bertola D, Hamanaka K, Fujita A, Mitsuhashi S, Miyatake S, Takata A, Miyake N, Mizuguchi T, Matsumoto N (2019). Primary immunodeficiency with chronic enteropathy and developmental delay in a boy arising from a novel homozygous RIPK1 variant. J Hum Genet.

[95] Lin L, Wang Y, Liu L, Ying W, Wang W, Sun B, Sun J, Wang X (2020). Clinical phenotype of a Chinese patient with RIPK1 deficiency due to novel mutation. Genes Dis.

[96] Sultan M, Adawi M, Kol N, McCourt B, Adawi I, Baram L, Tal N, Werner L, Lev A, Snapper SB, Barel O, Konnikova L, Somech R, Shouval DS (2022). RIPK1 mutations causing infantile-onset IBD with inflammatory and fistulizing features. Front Immunol.

[97] Tao P, Sun J, Wu Z, Wang S, Wang J, Li W, Pan H, Bai R, Zhang J, Wang Y, Lee PY, Ying W, Zhou Q, Hou J, Wang W, Sun B, Yang M, Liu D, Fang R, Han H, Yang Z, Huang X, Li H, Deuitch N, Zhang Y, Dissanayake D, Haude K, McWalter K, Roadhouse C, MacKenzie JJ, Laxer RM, Aksentijevich I, Yu X, Wang X, Yuan J, Zhou Q (2020). A dominant autoinflammatory disease caused by non-cleavable variants of RIPK1. Nature.

[98] Tapiz IRAJ, Cochino AV, Martins AL, Angosto-Bazarra D, de Landazuri IO, Mensa-Vilaro A, Cabral M, Baroja-Mazo A, Banos MC, Lobato-Salinas Z, Fabregat V, Plaza S, Yague J, Casals F, Oliva B, Figueiredo AE, Pelegrin P, Arostegui JI (2022). Characterization of novel pathogenic variants leading to caspase-8 cleavage-resistant RIPK1-induced autoinflammatory syndrome. J Clin Immunol.

[99] Kim SK, Kim WJ, Yoon JH, Ji JH, Morgan MJ, Cho H, Kim YC, Kim YS (2015). Upregulated RIP3 expression potentiates MLKL phosphorylation-mediated programmed necrosis in toxic epidermal necrolysis. J Investig Dermatol.

[100] Panayotova-Dimitrova D, Feoktistova M, Leverkus M (2015). RIPping the skin apart: necroptosis signaling in toxic epidermal necrolysis. J Investig Dermatol.

[101] Stadler PC, Clanner-Engelshofen BM, Helbig D, Satoh T, Reinholz M, French LE (2021). Necroptotic and apoptotic cell death in toxic epidermal necrolysis. J Dermatol Sci.

[102] Saito N, Qiao H, Yanagi T, Shinkuma S, Nishimura K, Suto A, Fujita Y, Suzuki S, Nomura T, Nakamura H, Nagao K, Obuse C, Shimizu H, Abe R (2014). An annexin A1-FPR1 interaction contributes to necroptosis of keratinocytes in severe cutaneous adverse drug reactions. Sci Transl Med.

[103] Duan X, Liu X, Liu N, Huang Y, Jin Z, Zhang S, Ming Z, Chen H (2020). Inhibition of keratinocyte necroptosis mediated by RIPK1/RIPK3/MLKL provides a protective effect against psoriatic inflammation. Cell Death Dis.

[104] Weisel K, Berger S, Papp K, Maari C, Krueger JG, Scott N, Tompson D, Wang S, Simeoni M, Bertin J, Peter Tak P (2020). Response to inhibition of receptor-interacting protein kinase 1 (RIPK1) in active plaque psoriasis: a randomized placebo-controlled study. Clin Pharmacol Ther.

[105] Saito N, Honma M, Shibuya T, Iinuma S, Igawa S, Kishibe M, Ishida-Yamamoto A (2018). RIPK1 downregulation in keratinocyte enhances TRAIL signaling in psoriasis. J Dermatol Sci.

[106] Witt A, Vucic D (2017). Diverse ubiquitin linkages regulate RIP kinases-mediated inflammatory and cell death signaling. Cell Death Differ.

[107] Bahr C, Rohwer A, Stempka L, Rincke G, Marks F, Gschwendt M (2000). DIK, a novel protein kinase that interacts with protein kinase Cdelta. Cloning, characterization, and gene analysis. J Biol Chem.

[108] Chen L, Haider K, Ponda M, Cariappa A, Rowitch D, Pillai S (2001). Protein kinase C-associated kinase (PKK), a novel membrane-associated, ankyrin repeat-containing protein kinase. J Biol Chem.

[109] Chen L, Hayden MS, Gilmore ES, Alexander-Savino C, Oleksyn D, Gillespie K, Zhao J, Poligone B (2017). PKK deletion in basal keratinocytes promotes tumorigenesis after chemical carcinogenesis. Carcinogenesis.

[110] Holland P, Willis C, Kanaly S, Glaccum M, Warren A, Charrier K, Murison J, Derry J, Virca G, Bird T, Peschon J (2002). RIP4 is an ankyrin repeat-containing kinase essential for keratinocyte differentiation. Curr Biol.

[111] Lee P, Jiang S, Li Y, Yue J, Gou X, Chen SY, Zhao Y, Schober M, Tan M, Wu X (2017). Phosphorylation of Pkp1 by RIPK4 regulates epidermal differentiation and skin tumorigenesis. EMBO J.

[112] Oberbeck N, Pham VC, Webster JD, Reja R, Huang CS, Zhang Y, Roose-Girma M, Warming S, Li Q, Birnberg A, Wong W, Sandoval W, Komuves LG, Yu K, Dugger DL, Maltzman A, Newton K, Dixit VM (2019). The RIPK4-IRF6 signalling axis safeguards epidermal differentiation and barrier function. Nature.

[113] Rountree RB, Willis CR, Dinh H, Blumberg H, Bailey K, Dean C, Peschon JJ, Holland PM (2010). RIP4 regulates epidermal differentiation and cutaneous inflammation. J Investig Dermatol.

[114] Huang CS, Oberbeck N, Hsiao YC, Liu P, Johnson AR, Dixit VM, Hymowitz SG (2018). Crystal structure of RIPK4 reveals dimerization-dependent kinase activity. Structure.

[115] Tanghe G, Urwyler-Rosselet C, De Groote P, Dejardin E, De Bock PJ, Gevaert K, Vandenabeele P, Declercq W (2018). RIPK4 activity in keratinocytes is controlled by the SCF(beta-TrCP) ubiquitin ligase to maintain cortical actin organization. Cell Mol Life Sci CMLS.

[116] Kim SW, Schifano M, Oleksyn D, Jordan CT, Ryan D, Insel R, Zhao J, Chen L (2014). Protein kinase C-associated kinase regulates NF-kappaB activation through inducing IKK activation. Int J Oncol.

[117] Muto A, Ruland J, McAllister-Lucas LM, Lucas PC, Yamaoka S, Chen FF, Lin A, Mak TW, Nunez G, Inohara N (2002). Protein kinase C-associated kinase (PKK) mediates Bcl10-independent NF-kappa B activation induced by phorbol ester. J Biol Chem.

[118] Kwa MQ, Scholz GM, Reynolds EC (2016). RIPK4 activates an IRF6-mediated proinflammatory cytokine response in keratinocytes. Cytokine.

[119] Adams S, Pankow S, Werner S, Munz B (2007). Regulation of NF-kappaB activity and keratinocyte differentiation by the RIP4 protein: implications for cutaneous wound repair. J Investig Dermatol.

[120] Poligone B, Gilmore ES, Alexander CV, Oleksyn D, Gillespie K, Zhao J, Ibrahim SF, Pentland AP, Brown MD, Chen L (2015). PKK suppresses tumor growth and is decreased in squamous cell carcinoma of the skin. J Investig Dermatol.

[121] Bae HC, Jeong SH, Kim JH, Lee H, Ryu WI, Kim MG, Son ED, Lee TR, Son SW (2018). RIP4 upregulates CCL20 expression through STAT3 signalling in cultured keratinocytes. Exp Dermatol.

[122] Moran ST, Haider K, Ow Y, Milton P, Chen L, Pillai S (2003). Protein kinase C-associated kinase can activate NFkappaB in both a kinase-dependent and a kinase-independent manner. J Biol Chem.

[123] Efimova T, Eckert RL (2000). Regulation of human involucrin promoter activity by novel protein kinase C isoforms. J Biol Chem.

[124] Papp H, Czifra G, Lazar J, Gonczi M, Csernoch L, Kovacs L, Biro T (2003). Protein kinase C isozymes regulate proliferation and high cell density-mediated differentiation in HaCaT keratinocytes. Exp Dermatol.

[125] Huang X, McGann JC, Liu BY, Hannoush RN, Lill JR, Pham V, Newton K, Kakunda M, Liu J, Yu C, Hymowitz SG, Hongo JA, Wynshaw-Boris A, Polakis P, Harland RM, Dixit VM (2013). Phosphorylation of Dishevelled by protein kinase RIPK4 regulates Wnt signaling. Science.

[126] Yi Z, Pu Y, Gou R, Chen Y, Ren X, Liu W, Dong P (2020). Silencing of RIPK4 inhibits epithelial-mesenchymal transition by inactivating the Wnt/beta-catenin signaling pathway in osteosarcoma. Mol Med Rep.

[127] Yu N, Kakunda M, Pham V, Lill JR, Du P, Wongchenko M, Yan Y, Firestein R, Huang X (2015). HSP105 recruits protein phosphatase 2A to dephosphorylate beta-catenin. Mol Cell Biol.

[128] Liu JY, Zeng QH, Cao PG, Xie D, Chen X, Yang F, He LY, Dai YB, Li JJ, Liu XM, Zeng HL, Zhu YX, Gong L, Cheng Y, Zhou JD, Hu J, Bo H, Xu ZZ, Cao K (2018). RIPK4 promotes bladder urothelial carcinoma cell aggressiveness by upregulating VEGF-A through the NF-kappaB pathway. Br J Cancer.

[129] Fuchs E (2007). Scratching the surface of skin development. Nature.

[130] Lim X, Nusse R (2013). Wnt signaling in skin development, homeostasis, and disease. Cold Spring Harb Perspect Biol.

[131] Reddy S, Andl T, Bagasra A, Lu MM, Epstein DJ, Morrisey EE, Millar SE (2001). Characterization of Wnt gene expression in developing and postnatal hair follicles and identification of Wnt5a as a target of Sonic hedgehog in hair follicle morphogenesis. Mech Dev.

[132] Reddy ST, Andl T, Lu MM, Morrisey EE, Millar SE (2004). Expression of Frizzled genes in developing and postnatal hair follicles. J Investig Dermatol.

[133] Millar SE, Willert K, Salinas PC, Roelink H, Nusse R, Sussman DJ, Barsh GS (1999). WNT signaling in the control of hair growth and structure. Dev Biol.

[134] Huelsken J, Vogel R, Erdmann B, Cotsarelis G, Birchmeier W (2001). Beta-Catenin controls hair follicle morphogenesis and stem cell differentiation in the skin. Cell.

[135] van Amerongen R, Berns A (2006). Knockout mouse models to study Wnt signal transduction. Trends Genet.

[136] Bhat AA, Sharma A, Pope J, Krishnan M, Washington MK, Singh AB, Dhawan P (2012). Caudal homeobox protein Cdx-2 cooperates with Wnt pathway to regulate claudin-1 expression in colon cancer cells. PLoS One.

[137] Miwa N, Furuse M, Tsukita S, Niikawa N, Nakamura Y, Furukawa Y (2001). Involvement of claudin-1 in the beta-catenin/Tcf signaling pathway and its frequent upregulation in human colorectal cancers. Oncol Res.

[138] Yan Y, Gauthier MA, Malik A, Fotiadou I, Ostrovski M, Dervovic D, Ghadban L, Tsai R, Gish G, Loganathan SK, Schramek D (2023). The NOTCH-RIPK4-IRF6-ELOVL4 axis suppresses squamous cell carcinoma. Cancers (Basel).

[139] Kwa MQ, Huynh J, Aw J, Zhang L, Nguyen T, Reynolds EC, Sweet MJ, Hamilton JA, Scholz GM (2014). Receptor-interacting protein kinase 4 and interferon regulatory factor 6 function as a signaling axis to regulate keratinocyte differentiation. J Biol Chem.

[140] Scholz GM, Sulaiman NS, Al Baiiaty S, Kwa MQ, Reynolds EC (2016). A novel regulatory relationship between RIPK4 and ELF3 in keratinocytes. Cell Signal.

[141] de la Garza G, Schleiffarth JR, Dunnwald M, Mankad A, Weirather JL, Bonde G, Butcher S, Mansour TA, Kousa YA, Fukazawa CF, Houston DW, Manak JR, Schutte BC, Wagner DS, Cornell RA (2013). Interferon regulatory factor 6 promotes differentiation of the periderm by activating expression of Grainyhead-like 3. J Invest Dermatol.

[142] Mitchell K, O'Sullivan J, Missero C, Blair E, Richardson R, Anderson B, Antonini D, Murray JC, Shanske AL, Schutte BC, Romano RA, Sinha S, Bhaskar SS, Black GC, Dixon J, Dixon MJ (2012). Exome sequence identifies RIPK4 as the Bartsocas–Papas syndrome locus. Am J Hum Genet.

[143] Kalay E, Sezgin O, Chellappa V, Mutlu M, Morsy H, Kayserili H, Kreiger E, Cansu A, Toraman B, Abdalla EM, Aslan Y, Pillai S, Akarsu NA (2012). Mutations in RIPK4 cause the autosomal-recessive form of popliteal pterygium syndrome. Am J Hum Genet.

[144] Kondo S, Schutte BC, Richardson RJ, Bjork BC, Knight AS, Watanabe Y, Howard E, de Lima RL, Daack-Hirsch S, Sander A, McDonald-McGinn DM, Zackai EH, Lammer EJ, Aylsworth AS, Ardinger HH, Lidral AC, Pober BR, Moreno L, Arcos-Burgos M, Valencia C, Houdayer C, Bahuau M, Moretti-Ferreira D, Richieri-Costa A, Dixon MJ, Murray JC (2002). Mutations in IRF6 cause Van der Woude and popliteal pterygium syndromes. Nat Genet.

[145] Lahtela J, Nousiainen HO, Stefanovic V, Tallila J, Viskari H, Karikoski R, Gentile M, Saloranta C, Varilo T, Salonen R, Kestila M (2010). Mutant CHUK and severe fetal encasement malformation. N Engl J Med.

[146] Biggs LC, Rhea L, Schutte BC, Dunnwald M (2012). Interferon regulatory factor 6 is necessary, but not sufficient, for keratinocyte differentiation. J Investig Dermatol.

[147] Ingraham CR, Kinoshita A, Kondo S, Yang B, Sajan S, Trout KJ, Malik MI, Dunnwald M, Goudy SL, Lovett M, Murray JC, Schutte BC (2006). Abnormal skin, limb and craniofacial morphogenesis in mice deficient for interferon regulatory factor 6 (Irf6). Nat Genet.

[148] Kwa MQ, Huynh J, Reynolds EC, Hamilton JA, Scholz GM (2015). Disease-associated mutations in IRF6 and RIPK4 dysregulate their signalling functions. Cell Signal.

[149] Richardson RJ, Dixon J, Malhotra S, Hardman MJ, Knowles L, Boot-Handford RP, Shore P, Whitmarsh A, Dixon MJ (2006). Irf6 is a key determinant of the keratinocyte proliferation-differentiation switch. Nat Genet.

[150] Hammond NL, Dixon J, Dixon MJ (2019). Periderm: life-cycle and function during orofacial and epidermal development. Semin Cell Dev Biol.

[151] Busa T, Jeraiby M, Clemenson A, Manouvrier S, Granados V, Philip N, Touraine R (2017). Confirmation that RIPK4 mutations cause not only Bartsocas–Papas syndrome but also CHAND syndrome. Am J Med Genet A.

[152] Gollasch B, Basmanav FB, Nanda A, Fritz G, Mahmoudi H, Thiele H, Wehner M, Wolf S, Altmuller J, Nurnberg P, Frank J, Betz RC (2015). Identification of a novel mutation in RIPK4 in a kindred with phenotypic features of Bartsocas–Papas and CHAND syndromes. Am J Med Genet A.

[153] Gripp KW, Ennis S, Napoli J (2013). Exome analysis in clinical practice: expanding the phenotype of Bartsocas–Papas syndrome. Am J Med Genet A.

[154] Leslie EJ, O'Sullivan J, Cunningham ML, Singh A, Goudy SL, Ababneh F, Alsubaie L, Ch'ng GS, van der Laar IM, Hoogeboom AJ, Dunnwald M, Kapoor S, Jiramongkolchai P, Standley J, Manak JR, Murray JC, Dixon MJ (2015). Expanding the genetic and phenotypic spectrum of popliteal pterygium disorders. Am J Med Genet A.

[155] Li Y, Tang L, Yue J, Gou X, Lin A, Weatherbee SD, Wu X (2020). Regulation of epidermal differentiation through KDF1-mediated deubiquitination of IKKalpha. EMBO Rep.

[156] Visscher MO, Carr AN, Narendran V (2022). Epidermal immunity and function: origin in neonatal skin. Front Mol Biosci.

[157] Candi E, Terrinoni A, Rufini A, Chikh A, Lena AM, Suzuki Y, Sayan BS, Knight RA, Melino G (2006). p63 is upstream of IKK alpha in epidermal development. J Cell Sci.

[158] Dohn M, Zhang S, Chen X (2001). p63alpha and DeltaNp63alpha can induce cell cycle arrest and apoptosis and differentially regulate p53 target genes. Oncogene.

[159] Moretti F, Marinari B, Lo Iacono N, Botti E, Giunta A, Spallone G, Garaffo G, Vernersson-Lindahl E, Merlo G, Mills AA, Ballaro C, Alema S, Chimenti S, Guerrini L, Costanzo A (2010). A regulatory feedback loop involving p63 and IRF6 links the pathogenesis of 2 genetically different human ectodermal dysplasias. J Clin Investig.

[160] Shamseldin HE, Khalifa O, Binamer YM, Almutawa A, Arold ST, Zaidan H, Alkuraya FS (2017). KDF1, encoding keratinocyte differentiation factor 1, is mutated in a multigenerational family with ectodermal dysplasia. Hum Genet.

[161] Thomason HA, Zhou H, Kouwenhoven EN, Dotto GP, Restivo G, Nguyen BC, Little H, Dixon MJ, van Bokhoven H, Dixon J (2010). Cooperation between the transcription factors p63 and IRF6 is essential to prevent cleft palate in mice. J Clin Investig.

[162] Westfall MD, Mays DJ, Sniezek JC, Pietenpol JA (2003). The Delta Np63 alpha phosphoprotein binds the p21 and 14-3-3 sigma promoters in vivo and has transcriptional repressor activity that is reduced by Hay-Wells syndrome-derived mutations. Mol Cell Biol.

[163] Xu J, Wei Q, He Z (2020). Insight into the function of RIPK4 in keratinocyte differentiation and carcinogenesis. Front Oncol.

[164] Liu DQ, Li FF, Zhang JB, Zhou TJ, Xue WQ, Zheng XH, Chen YB, Liao XY, Zhang L, Zhang SD, Hu YZ, Jia WH (2015). Increased RIPK4 expression is associated with progression and poor prognosis in cervical squamous cell carcinoma patients. Sci Rep.

[165] Peng K, Lin M, Wei Q, Li H, Zhang C, Xie R, Liu Z (2015). Association between RIPK4 relative copy number and prognosis of colorectal cancer patient after oxaliplatin-based chemotherapy. Zhonghua Wei Chang Wai Ke Za Zhi.

[166] Yi H, Su YZ, Lin R, Zheng XQ, Pan D, Lin DM, Gao X, Zhang R (2021). Downregulation of RIPK4 expression inhibits epithelial–mesenchymal transition in ovarian cancer through IL-6. J Immunol Res.

[167] Kim SW, Oleksyn DW, Rossi RM, Jordan CT, Sanz I, Chen L, Zhao J (2008). Protein kinase C-associated kinase is required for NF-kappaB signaling and survival in diffuse large B-cell lymphoma cells. Blood.

[168] Qi ZH, Xu HX, Zhang SR, Xu JZ, Li S, Gao HL, Jin W, Wang WQ, Wu CT, Ni QX, Yu XJ, Liu L (2018). RIPK4/PEBP1 axis promotes pancreatic cancer cell migration and invasion by activating RAF1/MEK/ERK signaling. Int J Oncol.

[169] Kopparam J, Chiffelle J, Angelino P, Piersigilli A, Zangger N, Delorenzi M, Meylan E (2017). RIP4 inhibits STAT3 signaling to sustain lung adenocarcinoma differentiation. Cell Death Differ.

[170] Gillison ML, Akagi K, Xiao W, Jiang B, Pickard RKL, Li J, Swanson BJ, Agrawal AD, Zucker M, Stache-Crain B, Emde AK, Geiger HM, Robine N, Coombes KR, Symer DE (2019). Human papillomavirus and the landscape of secondary genetic alterations in oral cancers. Genome Res.

[171] Li XC, Wang MY, Yang M, Dai HJ, Zhang BF, Wang W, Chu XL, Wang X, Zheng H, Niu RF, Zhang W, Chen KX (2018). A mutational signature associated with alcohol consumption and prognostically significantly mutated driver genes in esophageal squamous cell carcinoma. Ann Oncol.

[172] Li YY, Hanna GJ, Laga AC, Haddad RI, Lorch JH, Hammerman PS (2015). Genomic analysis of metastatic cutaneous squamous cell carcinoma. Clin Cancer Res.

[173] Pickering CR, Zhou JH, Lee JJ, Drummond JA, Peng SA, Saade RE, Tsai KY, Curry JL, Tetzlaff MT, Lai SY, Yu J, Muzny DM, Doddapaneni H, Shinbrot E, Covington KR, Zhang J, Seth S, Caulin C, Clayman GL, El-Naggar AK, Gibbs RA, Weber RS, Myers JN, Wheeler DA, Frederick MJ (2014). Mutational landscape of aggressive cutaneous squamous cell carcinoma. Clin Cancer Res.

[174] Stransky N, Egloff AM, Tward AD, Kostic AD, Cibulskis K, Sivachenko A, Kryukov GV, Lawrence MS, Sougnez C, McKenna A, Shefler E, Ramos AH, Stojanov P, Carter SL, Voet D, Cortes ML, Auclair D, Berger MF, Saksena G, Guiducci C, Onofrio RC, Parkin M, Romkes M, Weissfeld JL, Seethala RR, Wang L, Rangel-Escareno C, Fernandez-Lopez JC, Hidalgo-Miranda A, Melendez-Zajgla J, Winckler W, Ardlie K, Gabriel SB, Meyerson M, Lander ES, Getz G, Golub TR, Garraway LA, Grandis JR (2011). The mutational landscape of head and neck squamous cell carcinoma. Science.

[175] Loganathan SK, Schleicher K, Malik A, Quevedo R, Langille E, Teng K, Oh RH, Rathod B, Tsai R, Samavarchi-Tehrani P, Pugh TJ, Gingras AC, Schramek D (2020). Rare driver mutations in head and neck squamous cell carcinomas converge on NOTCH signaling. Science.

[176] Rogers MF, Gaunt TR, Campbell C (2020). CScape-somatic: distinguishing driver and passenger point mutations in the cancer genome. Bioinformatics.

[177] Loganathan SK, Schramek D (2020). In vivo CRISPR screens reveal potent driver mutations in head and neck cancers. Mol Cell Oncol.

[178] Xu J, Wu D, Zhang B, Pan C, Guo Y, Wei Q (2022). Depletion of RIPK4 parallels higher malignancy potential in cutaneous squamous cell carcinoma. PeerJ.

[179] Heim D, Cornils K, Schulze K, Fehse B, Lohse AW, Brummendorf TH, Wege H (2015). Retroviral insertional mutagenesis in telomerase-immortalized hepatocytes identifies RIPK4 as novel tumor suppressor in human hepatocarcinogenesis. Oncogene.

[180] Wang X, Zhu W, Zhou Y, Xu W, Wang H (2014). RIPK4 is downregulated in poorly differentiated tongue cancer and is associated with migration/invasion and cisplatin-induced apoptosis. Int J Biol Mark.

[181] Yeboah GK, Lobanova ES, Brush RS, Agbaga MP (2021). Very long chain fatty acid-containing lipids: a decade of novel insights from the study of ELOVL4. J Lipid Res.

[182] Kim C, Pasparakis M (2014). Epidermal p65/NF-kappaB signalling is essential for skin carcinogenesis. EMBO Mol Med.

[183] Mayo MW, Wang CY, Cogswell PC, Rogers-Graham KS, Lowe SW, Der CJ, Baldwin AS (1997). Requirement of NF-kappaB activation to suppress p53-independent apoptosis induced by oncogenic Ras. Science.

[184] Biggs LC, Naridze RL, DeMali KA, Lusche DF, Kuhl S, Soll DR, Schutte BC, Dunnwald M (2014). Interferon regulatory factor 6 regulates keratinocyte migration. J Cell Sci.

[185] Hu Y, Baud V, Delhase M, Zhang P, Deerinck T, Ellisman M, Johnson R, Karin M (1999). Abnormal morphogenesis but intact IKK activation in mice lacking the IKKalpha subunit of IkappaB kinase. Science.

[186] Li Q, Lu Q, Hwang JY, Buscher D, Lee KF, Izpisua-Belmonte JC, Verma IM (1999). IKK1-deficient mice exhibit abnormal development of skin and skeleton. Genes Dev.

[187] Liu B, Xia X, Zhu F, Park E, Carbajal S, Kiguchi K, DiGiovanni J, Fischer SM, Hu Y (2008). IKKalpha is required to maintain skin homeostasis and prevent skin cancer. Cancer Cell.

[188] Park E, Zhu F, Liu B, Xia X, Shen J, Bustos T, Fischer SM, Hu Y (2007). Reduction in IkappaB kinase alpha expression promotes the development of skin papillomas and carcinomas. Can Res.

[189] Takeda K, Takeuchi O, Tsujimura T, Itami S, Adachi O, Kawai T, Sanjo H, Yoshikawa K, Terada N, Akira S (1999). Limb and skin abnormalities in mice lacking IKKalpha. Science.

[190] Gareus R, Huth M, Breiden B, Nenci A, Rosch N, Haase I, Bloch W, Sandhoff K, Pasparakis M (2007). Normal epidermal differentiation but impaired skin-barrier formation upon keratinocyte-restricted IKK1 ablation. Nat Cell Biol.

[191] Gordon WM, Zeller MD, Klein RH, Swindell WR, Ho H, Espetia F, Gudjonsson JE, Baldi PF, Andersen B (2014). A GRHL3-regulated repair pathway suppresses immune-mediated epidermal hyperplasia. J Clin Investig.

[192] Hislop NR, Caddy J, Ting SB, Auden A, Vasudevan S, King SL, Lindeman GJ, Visvader JE, Cunningham JM, Jane SM (2008). Grhl3 and Lmo4 play coordinate roles in epidermal migration. Dev Biol.

[193] Kashgari G, Venkatesh S, Refuerzo S, Pham B, Bayat A, Klein RH, Ramos R, Ta AP, Plikus MV, Wang PH, Andersen B (2021). GRHL3 activates FSCN1 to relax cell-cell adhesions between migrating keratinocytes during wound reepithelialization. JCI Insight.

[194] Ting SB, Caddy J, Wilanowski T, Auden A, Cunningham JM, Elias PM, Holleran WM, Jane SM (2005). The epidermis of grhl3-null mice displays altered lipid processing and cellular hyperproliferation. Organogenesis.

[195] Ting SB, Wilanowski T, Auden A, Hall M, Voss AK, Thomas T, Parekh V, Cunningham JM, Jane SM (2003). Inositol- and folate-resistant neural tube defects in mice lacking the epithelial-specific factor Grhl-3. Nat Med.

[196] Yu Z, Lin KK, Bhandari A, Spencer JA, Xu X, Wang N, Lu Z, Gill GN, Roop DR, Wertz P, Andersen B (2006). The Grainyhead-like epithelial transactivator Get-1/Grhl3 regulates epidermal terminal differentiation and interacts functionally with LMO4. Dev Biol.

[197] Lee S, Kong Y, Weatherbee SD (2013). Forward genetics identifies Kdf1/1810019J16Rik as an essential regulator of the proliferation-differentiation decision in epidermal progenitor cells. Dev Biol.

[198] Herron BJ, Liddell RA, Parker A, Grant S, Kinne J, Fisher JK, Siracusa LD (2005). A mutation in stratifin is responsible for the repeated epilation (Er) phenotype in mice. Nat Genet.

[199] Li Q, Lu Q, Estepa G, Verma IM (2005). Identification of 14–3-3sigma mutation causing cutaneous abnormality in repeated-epilation mutant mouse. Proc Natl Acad Sci USA.

[200] Richardson RJ, Hammond NL, Coulombe PA, Saloranta C, Nousiainen HO, Salonen R, Berry A, Hanley N, Headon D, Karikoski R, Dixon MJ (2014). Periderm prevents pathological epithelial adhesions during embryogenesis. J Clin Investig.

[201] Winter M, Lodygin D, Verdoodt B, Hermeking H (2016). Deletion of 14–3-3sigma sensitizes mice to DMBA/TPA-induced papillomatosis. Oncotarget.

